# SWEET11b transports both sugar and cytokinin in developing barley grains

**DOI:** 10.1093/plcell/koad055

**Published:** 2023-03-01

**Authors:** Volodymyr Radchuk, Zeinu M Belew, Andre Gündel, Simon Mayer, Alexander Hilo, Goetz Hensel, Rajiv Sharma, Kerstin Neumann, Stefan Ortleb, Steffen Wagner, Aleksandra Muszynska, Christoph Crocoll, Deyang Xu, Iris Hoffie, Jochen Kumlehn, Joerg Fuchs, Fritz F Peleke, Jedrzej J Szymanski, Hardy Rolletschek, Hussam H Nour-Eldin, Ljudmilla Borisjuk

**Affiliations:** Leibniz-Institute of Plant Genetics and Crop Plant Research (IPK), Corrensstrasse 3, 06466 Gatersleben, Germany; Faculty of Science, Department of Plant and Environmental Sciences, DynaMo Center of Excellence, University of Copenhagen, Thorvaldsensvej 40, 1871 Frederiksberg C, Denmark; Leibniz-Institute of Plant Genetics and Crop Plant Research (IPK), Corrensstrasse 3, 06466 Gatersleben, Germany; Leibniz-Institute of Plant Genetics and Crop Plant Research (IPK), Corrensstrasse 3, 06466 Gatersleben, Germany; Institute of Experimental Physics 5, University of Würzburg, Am Hubland, 97074 Würzburg, Germany; Leibniz-Institute of Plant Genetics and Crop Plant Research (IPK), Corrensstrasse 3, 06466 Gatersleben, Germany; Leibniz-Institute of Plant Genetics and Crop Plant Research (IPK), Corrensstrasse 3, 06466 Gatersleben, Germany; Centre of Region Haná for Biotechnological and Agricultural Research, Czech Advanced Technology and Research Institute, Palacký University Olomouc, 78371 Olomouc, Czech Republic; Scotland’s Rural College (SRUC), Kings Buildings, West Mains Road, Edinburgh, EH9 3JGUK; Leibniz-Institute of Plant Genetics and Crop Plant Research (IPK), Corrensstrasse 3, 06466 Gatersleben, Germany; Leibniz-Institute of Plant Genetics and Crop Plant Research (IPK), Corrensstrasse 3, 06466 Gatersleben, Germany; Leibniz-Institute of Plant Genetics and Crop Plant Research (IPK), Corrensstrasse 3, 06466 Gatersleben, Germany; Leibniz-Institute of Plant Genetics and Crop Plant Research (IPK), Corrensstrasse 3, 06466 Gatersleben, Germany; Faculty of Science, Department of Plant and Environmental Sciences, DynaMo Center of Excellence, University of Copenhagen, Thorvaldsensvej 40, 1871 Frederiksberg C, Denmark; Faculty of Science, Department of Plant and Environmental Sciences, DynaMo Center of Excellence, University of Copenhagen, Thorvaldsensvej 40, 1871 Frederiksberg C, Denmark; Leibniz-Institute of Plant Genetics and Crop Plant Research (IPK), Corrensstrasse 3, 06466 Gatersleben, Germany; Leibniz-Institute of Plant Genetics and Crop Plant Research (IPK), Corrensstrasse 3, 06466 Gatersleben, Germany; Leibniz-Institute of Plant Genetics and Crop Plant Research (IPK), Corrensstrasse 3, 06466 Gatersleben, Germany; Leibniz-Institute of Plant Genetics and Crop Plant Research (IPK), Corrensstrasse 3, 06466 Gatersleben, Germany; Leibniz-Institute of Plant Genetics and Crop Plant Research (IPK), Corrensstrasse 3, 06466 Gatersleben, Germany; IBG-4 Bioinformatics, Forschungszentrum Jülich, 52428 Jülich, Germany; Leibniz-Institute of Plant Genetics and Crop Plant Research (IPK), Corrensstrasse 3, 06466 Gatersleben, Germany; Faculty of Science, Department of Plant and Environmental Sciences, DynaMo Center of Excellence, University of Copenhagen, Thorvaldsensvej 40, 1871 Frederiksberg C, Denmark; Leibniz-Institute of Plant Genetics and Crop Plant Research (IPK), Corrensstrasse 3, 06466 Gatersleben, Germany

## Abstract

Even though Sugars Will Eventually be Exported Transporters (SWEETs) have been found in every sequenced plant genome, a comprehensive understanding of their functionality is lacking. In this study, we focused on the SWEET family of barley (*Hordeum vulgare*). A radiotracer assay revealed that expressing *HvSWEET11b* in African clawed frog (*Xenopus laevis*) oocytes facilitated the bidirectional transfer of not only just sucrose and glucose, but also cytokinin. Barley plants harboring a loss-of-function mutation of *HvSWEET11b* could not set viable grains, while the distribution of sucrose and cytokinin was altered in developing grains of plants in which the gene was knocked down. Sucrose allocation within transgenic grains was disrupted, which is consistent with the changes to the cytokinin gradient across grains, as visualized by magnetic resonance imaging and Fourier transform infrared spectroscopy microimaging. Decreasing *HvSWEET11b* expression in developing grains reduced overall grain size, sink strength, the number of endopolyploid endosperm cells, and the contents of starch and protein. The control exerted by HvSWEET11b over sugars and cytokinins likely predetermines their synergy, resulting in adjustments to the grain's biochemistry and transcriptome.

## Teaser

SWEET family proteins can additionally transport cytokinins synergistically with sugars during barley grain filling

**IN A NUTSHELL**

**Background:** Sugars Will Eventually be Exported Transporter (SWEET) is a large family of proteins, which have been found in every sequenced plant genome. The main function of SWEET proteins is the transport of sugars like sucrose and glucose. This makes SWEETs important for various processes during a plant's growth and development. However, the SWEET family in barley was not characterized so far either in terms of its capabilities to transport specific substrates or their functional roles in grain's growth and development.

**Questions:** What is the physiological role of SWEETs in barley grain development? Which substrates are transported by the SWEET proteins present in the seed?

**Findings:** Of 23 barley SWEET genes, *HvSWEET11b*, *HvSWEET15a*, and *HvSWEET4* are predominantly active in the developing grains. HvSWEET11b protein functions not only as a sugar transporter but is able also to transport the phytohormone cytokinin. Plants carrying a knockout homozygous mutation of *HvSWEET11b* failed to set any viable grain. The partial repression of *HvSWEET11b* transcription altered the allocation of both sucrose and cytokinin in the grain and resulted in fewer endosperm cells, lower starch and protein accumulation, and a reduction of the grain size at maturity. The dual substrate capacity of a single transporter protein provides the plant with an efficient means of coordinating the grain's development and filling.

**Next steps:** This study highlights the relevance of SWEET proteins as multifunctional transporters. It would be of great interest to explore the molecular mechanisms of how cytokinin transport and metabolism toward and in the grains influence their development.

## Introduction

Plants synthesize many molecules that must be moved between cells and organs. When this movement involves passage through a semipermeable membrane, proteins (described as transporters, facilitators, or carriers) are used to control the process. Although plant genomes encode many such proteins, their number is far smaller than the number of compounds that are transported, so many transporters have evolved multifunctionality. For example, members of the Purine Permease (PUP) family have been implicated in the transport of not only purine nucleobases (Gillissen et al. 2000) along with derivatives such as cytokinin (Kang et al. 2017) but also nonpurine secondary metabolites (Jelesko 2012). Nitrate transporter/peptide transporter family proteins can transport single and dipeptides, nitrate, nitrite, chloride, glucosinolate, and various phytohormones and secondary metabolites (Krouk et al. 2010; Kanno et al. 2012; Nour-Eldin et al. 2012; Saito et al. 2015; Corratgé-Faillie and Lacombe 2017; Michniewicz et al. 2019; Kazachkova et al. 2021). The *Arabidopsis thaliana* protein AtABCG1 (G-type ATP BINDING CASSETTE TRANSPORTER 1) mediates the transport of lipidic and phenolic compounds, dicarboxylic acids, fatty alcohols, fatty acids, and auxin (Yim et al. 2016; Shanmugarajah et al. 2019; Liu et al. 2020), whereas the plant uses AtABCG14 to transport squalene-derived metabolites and cytokinins (Le Hir et al. 2013; Ko et al. 2014; Zhang et al. 2014). The system used to transport phytohormones is highly redundant, with multiple transporters available for a given phytohormone (Anfang and Shani 2021). Certain *A. thaliana* Sugars Will Eventually be Exported Transporter (SWEET) proteins, initially considered to facilitate the transport of sucrose (Chen et al. 2015a; Han et al. 2017), have since been shown to also be involved in the movement of gibberellins (Kanno et al. 2016). Similarly, the rice (*Oryza sativa*) protein OsSWEET3a acts as a transporter of gibberellin and glucose (Morii et al. 2020).

SWEET family members are responsible for various processes during plant growth and development (Chen et al. 2015b; Wang et al. 2019, 2020; Breia et al. 2021). Throughout seed development, the filial component remains encased by maternal tissue. The mother plant supplies the compounds required by the filial tissues, which must then be transported across the maternal–filial boundary (Radchuk and Borisjuk 2014). The products of *A. thaliana SWEET11*, *SWEET12*, and *SWEET15*, each active in the seed coat, are required to transport sucrose from the mother plant into the developing embryo (Chen et al. 2015b). Similarly, in soybean (*Glycine max*), GmSWEET10a and GmSWEET10b are present in the seed coat, where they transport mono- and disaccharides into the developing endosperm and embryo (Wang et al. 2020). The rice genes *OsSWEET11* and *OsSWEET15*, both of which encode sucrose transporters, are both transcribed in the nucellus, vascular bundle, nucellar projection, nucellar epidermis, and aleurone (Ma et al. 2017; Yang et al. 2018). Double knockout *ossweet11; 15* mutants lack functional endosperm, leading to the identification of these genes as key players in seed filling in rice (Yang et al. 2018). OsSWEET14, in cooperation with OsSWEET11, also contributes to grain filling in rice (Fei et al. 2021). Maize (*Zea mays*) ZmSWEET4c and rice OsSWEET4 can transfer hexoses and operate within filial but not maternal tissue; in both cases, loss-of-function mutants are severely compromised in the formation of viable endosperm (Sosso et al. 2015).

In both barley (*Hordeum vulgare*) and wheat (*Triticum aestivum*), most if not all of the sucrose delivered to the endosperm moves through a specialized assimilate allocation route (Melkus et al. 2011) comprising the vascular bundle and nucellar projection on the maternal side, endosperm transfer cells on the filial side, and the apoplastic space in-between. Disturbing the structure and/or function of the nucellar projection has a major detrimental effect on the effectiveness of the assimilate transfer process (Radchuk et al. 2006, 2021). Several transporters, including sucrose transporters (SUTs), which move sucrose across the plasma membrane from the apoplast, are active in the nucellar projection and endosperm transfer cells (Thiel et al. 2008). In the barley grain, the plasma membrane-localized protein HvSUT1 is present in endosperm transfer cells and the nucellar projection, where it controls the balance of sucrose along the delivery path in conjunction with the vacuole-based HvSUT2 (Radchuk et al. 2017). The transcription of *HvSWEET11b* is largely restricted to the nucellar projection (Mascher et al. 2017), making it a likely partner of HvSUT1. However, the precise function of this SWEET protein—and indeed, that of other barley SWEETs—is currently unclear.

Here, our study of the functions of different SWEET proteins in barley grains during the grain filling process revealed 2 major findings. First, we identified the key players in sucrose uptake in grains during seed filling, and second, we determined that SWEET proteins are able to transport cytokinin.

## Results

### SWEET genes are differentially expressed in the developing barley grain

**Figure 1.**
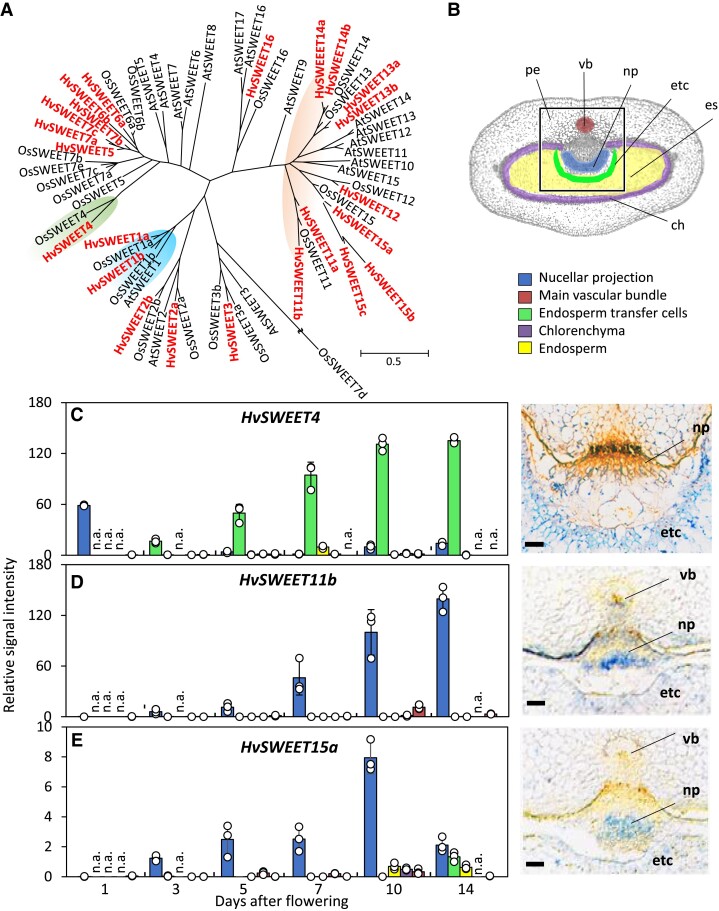
The *SWEET* gene family is in barley. **A**) The phylogeny of SWEET polypeptides encoded by the *H. vulgare* (Hv) (shown in red), *O. sativa* (Os), and *A. thaliana* (At) genomes. One thousand bootstrap replicates support the tree. The scale bar represents evolutionary distances, as quantified by the number of substitutions per amino acid. Gene IDs for barley and rice are given in Supplemental Data Set S8, while those for *A. thaliana* are taken from Chen et al. (2010). **B**) Grain cross-section indicating the sites where laser micro-dissected tissues were sampled for RT-qPCR. The tissue color code corresponds to the bars in (**C**–**E**). The white square indicates the regions assayed by in situ hybridizations, shown in (C–E, right). Tissue-specific expression of *HvSWEET4* (**C**), *HvSWEET11b* (**D**), and *HvSWEET1* (**E**), as revealed by RT-qPCR of micro-dissected tissues (left) and in situ hybridization (right). RT-qPCR data are given as the mean ± standard deviation (Sd) (*n* = 3 biological replicates, each derived from microdissection of 3 caryopses; individual samples are shown as dots). Bars: 100 *µ*m. ch, chlorenchyma; es, endosperm; etc, endosperm transfer cells; n.a., not analyzed; np, nucellar projection; pe, pericarp; vb, vascular bundle.

Ten of the 23 *SWEET* sequences present in the barley genome (Mascher et al. 2017) belong to a clade of genes likely encoding SUTs (Fig. 1A). Unlike their putative orthologs in rice (Yuan et al. 2014) and *A. thaliana* (Chen et al. 2012)*, SWEET11*, *SWEET13*, *SWEET14*, and *SWEET15* are each represented in barley by multiple (2 or 3) genes rather than by a single copy. In addition, the barley genome harbors 1 *HvSWEET4* gene likely encoding a hexose transporter and 2 genes (*HvSWEET1a* and *HvSWEET1b*) likely encoding glucose transporters. We performed an RT-qPCR assay to quantify the transcription of *HvSWEET4* and *HvSWEET11*–*HvSWEET15* throughout development. The assays failed to reveal the presence of *HvSWEET12*, *HvSWEET13a*, *HvSWEET13b*, *HvSWEET15b*, or *HvSWEET15c* transcripts but did detect low levels of *HvSWEET11a*, *HvSWEET14a*, and *HvSWEET14b* transcripts (Supplemental Fig. S1). *HvSWEET4*, *HvSWEET11b*, and *HvSWEET15a* displayed similar temporal patterns of transcription: their transcripts were highly abundant, particularly during the main filling stage. A more detailed analysis of these 3 genes, based on both RT-qPCR profiling of microdissected grain tissue (Fig. 1B) and in situ hybridization, revealed that *HvSWEET4* transcript accumulated in endosperm transfer cells (Fig. 1C); *HvSWEET11b* transcript accumulated in the nucellus and nucellar projection (and at low levels in the vascular bundle) (Fig. 1D); and *HvSWEET15a* transcript accumulated in the nucellar projection, although at 4- to 9-fold lower levels than *HvSWEET11b* (Fig. 1E). The highly similar temporal and spatial expression profiles of *HvSWEET11b* and *HvSWEET15a* point to a certain level of functional redundancy, at least in the nucellar projection. Low to moderate levels of both *HvSWEET4* and *HvSWEET15a* transcripts were present in vegetative and reproductive tissues, whereas *HvSWEET11b* transcription was almost exclusively limited to the developing grain (Supplemental Fig. S1B). The implication of the distinct difference in transcript abundance between the highly similar gene pairs *HvSWEET11a/HvSWEET11b* and *HvSWEET14a/HvSWEET14b* was that, following the duplication of their ancestral gene, a measure of neofunctionalization has occurred. Both *HvSWEET15b* and *HvSWEET15c* harbor a rather short open reading frame, and neither was transcribed in any of the tissues analyzed; it is likely that both are pseudogenes.

### Barley SWEETs exhibit distinct sucrose and glucose transport activities

**Figure 2.**
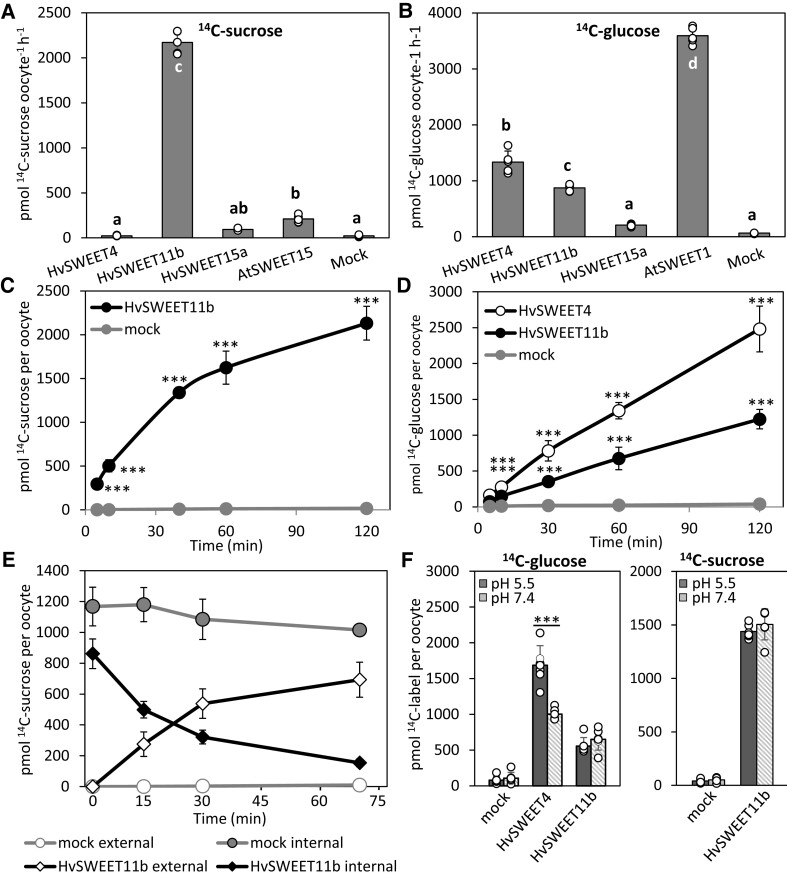
Functional analysis of HvSWEET4, HvSWEET11b, and HvSWEET15a in *Xenopus* oocytes. The ability of barley SWEETs to mediate the transport of sucrose and glucose was assayed by incubating *SWEET-*expressing oocytes in Kulori buffer (pH 7.4) containing 10 mM ^14^C-sucrose (**A**) or 10 mM ^14^C-glucose (**B**) for 1 h. All values are means ± Sd. Different letters indicate significant differences at *P* < 0.001 determined by 1-way ANOVA with the Bonferroni correction test (*n* = 5 biological replicates consisting of individual oocytes). Uptake of ^14^C-sucrose mediated by HvSWEET11b (**C**) and ^14^C-glucose mediated by HvSWEET11b and HvSWEET4 (**D**). The assays were done in Kulori buffer (pH 7.4) containing 10 mM ^14^C-sucrose (C) or 10 mM ^14^C-glucose (D). **E**) The export of ^14^C-sucrose mediated by HvSWEET11b following its injection into oocytes. **F**) The influence of pH on the ability of HvSWEET4 and HvSWEET11b to mediate the transport of glucose (left) and HvSWEET11b to mediate the transport of sucrose (right) in a 10-mM sugar uptake assay for 1 h. All values are means ± Sd (*n* = 6 biological replicates [oocytes] in C and F, *n* = 3 to 4 biological replicates [oocytes] in D and E). ***P < 0.001 as determined by 2-tailed Student's *t*-test between mock oocytes and oocytes expressing the corresponding protein. Individual samples are shown as dots in (A, B, F).

We investigated the transport activity and substrate specificity of HvSWEET4, HvSWEET11b, and HvSWEET15a via heterologous expression in African clawed frog (*Xenopus laevis*) oocytes using a radiotracer-based transport assay. The *A. thaliana* orthologs AtSWEET15 and AtSWEET1, which are associated with a high capacity to transport sucrose (Chen et al. 2012) and glucose (Chen et al. 2010), were chosen as positive controls. An analysis of ^14^C-labeled sucrose uptake revealed that HvSWEET11b transported sucrose 8 times more effectively than AtSWEET15 (Fig. 2A). HvSWEET15a had a weak ability to transport sucrose, while HvSWEET4 was unable to transport sucrose at all (Fig. 2A). A parallel analysis based on the uptake of ^14^C-labeled glucose showed that the uptake efficiency of HvSWEET4 was higher than that of HvSWEET11b but still much lower than that of AtSWEET1 (Fig. 2B). HvSWEET15a could not take up glucose. These and the following results were confirmed using independent batches of oocytes (Supplemental Fig. S2A). The uptake of sucrose by HvSWEET11b was detectable within 5 min of the cells being supplied with the substrate and increased linearly during the first ∼30 min, after which the uptake rate approached a plateau (Fig. 2C; Supplemental Fig. S2B). Concerning glucose, both HvSWEET4 and HvSWEET11b allowed the sugar to accumulate monotonically over time (Fig. 2D).

Because SWEETs have been characterized as bidirectional sugar transporters (Chen et al. 2012), we examined the ability of HvSWEET11b to export sucrose using an injection-based export assay (Fig. 2E). Following the injection of 23 nL 50 mM ^14^C-sucrose into oocytes expressing *HvSWEET11b*, ∼65% of the injected sucrose was released into the external medium over the first 30 min and ∼83% after 70 min. In contrast, after 70 min, only 7% of the injected sucrose was released from mock oocytes (injected with water instead of complementary RNA [cRNA]). The experiment confirmed that HvSWEET11b transports sucrose along the sucrose concentration gradient.

Changes in the pH of the medium did not influence the ability of HvSWEET11b to transport sucrose or glucose, but the glucose transport activity of HvSWEET4 was enhanced under acidic (pH 5.5) conditions (Fig. 2F). This increase in HvSWEET4-mediated glucose transport at acidic pH is unlikely to be due to the cotransport of protons, as it failed to generate a current in two-electrode voltage-clamp (TEVC) recording (Supplemental Fig. S3). The pH of the apoplastic fluid in front of the endosperm transfer cells, as measured using a pH microsensor, was ∼6.6. Therefore, HvSWEET11b functions as a bidirectional sugar transporter with very high sucrose and moderate glucose transport activity, while HvSWEET4 is an effective glucose transporter.

### Knock-down of *HvSWEET11b* compromises sucrose allocation in the developing grain

On the assumption that HvSWEET11b and HvSWEET15a are functionally redundant, we mutated both genes using clustered regularly interspaced short palindromic repeats/CRISPR-associated protein 9 (CRISPR-Cas9) (Supplemental Fig. S4A). Plants carrying a knockout mutation of *HvSWEET11b* in the homozygous state failed to set any viable grains, regardless of whether they also carried a knockout mutation in *HvSWEET15a* (Supplemental Fig. S4B). The vegetative growth and the spike formation of the mutants were indistinguishable from the wild type (WT). Analysis of florets revealed that *HvSWEET11b* was expressed in the anther and not in the gynoecium (Supplemental Fig. S4C). Homozygous *hvsweet11b* plants produced viable pollen (Supplemental Fig. S4, D and E) and could set seed (Supplemental Fig. S4F); the mutation in *HvSWEET11b* probably did not affect pollen development. Additionally, the vast majority (∼90%) of caryopses grew well during early development but stopped growing at the onset of the storage phase; at this point they became thinner compared to the WT, and all died during the early grain filling period (Supplemental Fig. S4, F and G). This phenotype may result from the inability of *hvsweet11b* caryopses to attract and/or accumulate nutrients. We conclude that HvSWEET11b plays pivotal roles in the filling capacity of barley grains and their survival.

**Figure 3.**
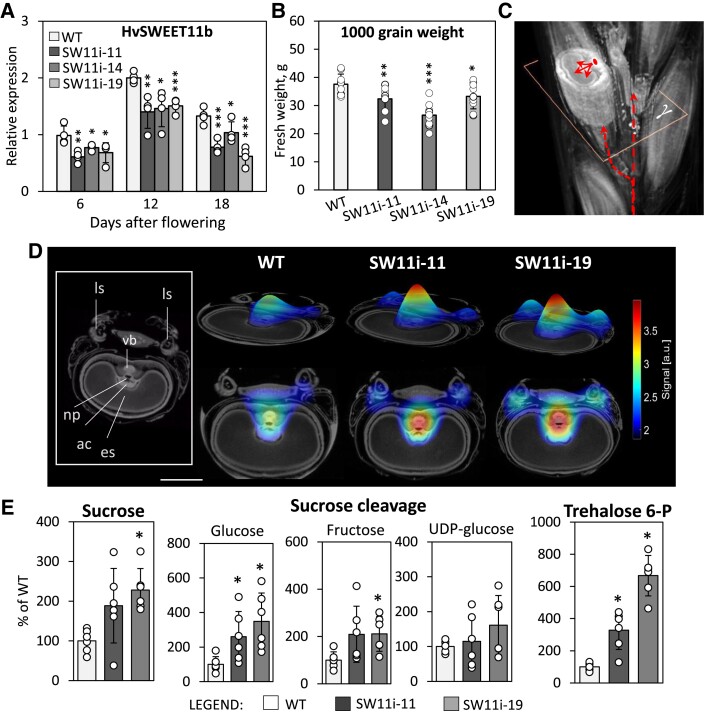
The effect of knocking down *HvSWEET11b* on sucrose allocation and carbon metabolism. **A**) Reduction in *HvSWEET11b* transcript levels in transgenic *HvSWEET11b*-repressed developing grains compared to the WT, as analyzed by an RT-qPCR assay. **B**) In the presence of the transgene, the weight of the mature grain is reduced. Values in (A) and (B) are means; error bars represent Sd; in (A): *n* = 4 biological replicates each consisting of 4 to 10 caryopses from 3 spikes, in (B): *n* = 9 biological replicates each consisting of ∼100 grains). *P < 0.05, **P < 0.01, and ***P < 0.001 as determined by 2-tailed Student's *t*-test between WT and the corresponding transgenic line. **C**) Virtual cross-section of a grain in a living spike, visualized by MRI, to monitor the distribution of ^13^C-labeled sucrose shown in (D). Red arrows indicate the direction of sucrose flow. **D**) The distribution of ^13^C-labeled sucrose, as captured by MRI in developing grains set by WT and 2 *SWEET11b* knockdown transgenic lines. The sucrose concentration is color-coded. The various tissues present in the grain are identified in the insert shown on the left. Scale bar = 1 mm. **E**) Abundance of sugars measured in developing caryopsis (12 DAF). Data are given as mean ± Sd and normalized to the WT level (=100%). *P < 0.05, as determined by the Mann–Whitney *U*-test between WT and the corresponding transgenic line (*n* = 6 biological replicates, each consisting of 4 to 10 caryopses from 3 spikes). Individual samples are shown as dots in (A, B, E). ac, apoplastic cavity; a.u., arbitrary units; es, endosperm; ls, lateral spikelet; np, nucellar projection; vb, vascular bundle.

To analyze the role of *HvSWEET11b* in grain development, we produced *HvSWEET11b*-repressed plants using RNAi-technology. Of the 17 independent transgenic lines, 11 plants were able to set viable grains (Supplemental Fig. S5). Three homozygous knockdown plant lines were selected for further analysis. As analyzed by RT-qPCR, a moderate suppression of *HvSWEET11b* transcription was achieved in these lines by the presence of the transgene (Fig. 3A). The abundance of both *HvSWEET11a* and *HvSWEET15a* transcripts in the grain was unaffected in the mutants (Supplemental Fig. S6). Similar to the *HvSWEET11b* knockout mutants, there was no discernible phenotypic effect of the gene knockdown on either vegetative development or spike formation. However, repressing *HvSWEET11b* expression caused a reduction in thousand grain weight (TGW) (Fig. 3B) and grain width (Supplemental Fig. S7).

We used a magnetic resonance imaging (MRI) platform (Melkus et al. 2011) to compare sucrose transport in planta at the start of the assimilate storage phase between WT and 2 independent *HvSWEET11b* knockdown plants. The developing grains were supplied with ^13^C-sucrose for 24 h, after which the distribution of the label was visualized in virtual cross-sections of the grain (Fig. 3, C and D). In both WT and transgenic grains, sucrose accumulated most strongly along the major sucrose transfer route, i.e. the vascular bundle and nucellar projection, as well as in the surrounding pericarp and endosperm cavity (Fig. 3D). However, not only was a higher degree of accumulation observed in transgenic grains, but there was also clear evidence for a greater level of sucrose deposition in both lateral sterile spikelets (Fig. 3D). We conclude that suppressing *HvSWEET11b* expression in the nucellar projection inhibited the release of sucrose from the tissue toward the endosperm, resulting in the build-up of sucrose in the maternal tissue component of the major transport route; the sucrose was then redirected toward alternative sinks such as lateral spikelets.

### The metabolite pattern of *HvSWEET11b*-knockdown grains does not show signs of carbon starvation

We characterized the metabolic consequences of knocking-down *HvSWEET11b* using an untargeted metabolomic approach. Of the 78 metabolites identified, 25 were significantly more abundant in knockdown grains than in WT grains, but only isocitrate was significantly less abundant in knockdown grains (Supplemental Data Set S1). The transgenic grains contained higher levels of sucrose and its cleavage products (glucose and fructose; Fig. 3E) than the WT, and notably, a greater content of the signaling sugar trehalose 6-phosphate. They also accumulated more phosphorylated hexoses than the WT (Supplemental Fig. S6C); these compounds fuel the majority of central metabolic pathways. A shift in the ratio of mono- to triphosphorylated nucleotides, a greater accumulation of cofactors (pantothenate, pyridoxal-5-phosphate), and an up to 6-fold higher level of the antioxidant ascorbate were observed in knockdown versus WT grains. This pattern may reflect a lower expenditure of carbon skeletons. Therefore, knocking down *HvSWEET11b* in developing grains shifted the balance between the supply and demand of carbon. Thus, the aberrant allocation of sucrose induced by knocking-down *HvSWEET11b* did not cause carbon starvation.

### The transcriptomic consequences of down-regulating *HvSWEET11b*

We performed transcriptome deep sequencing (RNA-seq) to compare the transcriptomes of 6- and 12-d-old grains of *HvSWEET11b*-knockdown and WT plants. At the time of the first sampling, 97 out of 20,995 genes appeared to be more abundantly transcribed in transgenic grains, whereas 115 were less abundantly transcribed in transgenic grains (Supplemental Data Set S2). At 12 d after flowering, only 15 and 12 genes were up- and downregulated, respectively, in the grains of *HvSWEET11b*-knockdown plants. Along with the downregulation of *HvSWEET11b*, the *HvSWEET14b* transcript level also decreased, while other genes encoding sugar-transporting proteins were unaffected in knockdown plants (Table 1). The transcript levels of *sucrose synthase 4*, encoding the major enzyme of sucrose cleavage, and 2 genes encoding AGPase, the rate-limiting enzyme in starch biosynthesis (Hannah and James 2008), were downregulated by the presence of the RNAi transgene, indicating impaired starch synthesis. Starch consists of the relatively simple polymer amylose, which is synthesized by granule-bound starch synthase (SS), and the rather complex polymer amylopectin, whose production requires soluble SS, starch-branching enzyme (SBE), and starch-debranching enzyme activities (Hannah and James 2008). The expression of endosperm-specific *SS2a*, *SBE3a*, and *SBE3b* (Radchuk et al. 2009) was downregulated in the transgenic grains. A gene encoding mannose-1-phosphate guanyltransferase, an enzyme required for synthesizing the cell wall polysaccharide mannan, was also repressed in transgenic grains. The list of the genes upregulated by the presence of the RNAi transgene included those encoding callose synthase, fructan 1-exohydrolase, mannan endo-1,4-β-mannosidase, and cellulose synthase (Table 1). No genes encoding either α- or β-amylase were upregulated in the transgenic grains. Thus, the induction of starch remobilization, a process known to occur in response to sucrose starvation (Radchuk et al. 2017; Chen et al. 2019), was not apparent in *HvSWEET11b*-repressed grains.

**Table 1.** Differential expression of genes involved in starch and protein biosynthesis as well as cytokinin signaling in *HvSWEET11*-repressed grains compared with WT

Gene ID	Gene description	6 DAF*	12 DAF*
Genes related to sugar conversion and transport			
HORVU4Hr1G037380	Cellulose synthase A3	**−2**.**46**	−0.71
HORVU7Hr1G054710	SWEET11b	**−2**.**22**	**−1**.**15**
HORVU6Hr1G000440	SWEET14b	**−1**.**75**	**−1**.**40**
HORVU3Hr1G014090	NAC domain protein 019	**−1**.**33**	−0.33
HORVU7Hr1G033230	Sucrose synthase 4	**−1**.**20**	−0.39
HORVU7Hr1G067620	Glucose-1P adenylyltransferase (AGPase)	**−1**.**25**	−0.23
HORVU1Hr1G091600	Large subunit L1 of AGPase	**−1**.**27**	0.26
HORVU7Hr1G038420	Starch synthase 2a	**−1**.**04**	0.22
HORVU0Hr1G022780	Starch branching enzyme 3a	**−1**.**09**	−0.15
HORVU0Hr1G022790	Starch branching enzyme 3b	**−2**.**11**	−0.37
HORVU4Hr1G060380	Mannose-1-phosphate guanyltransferase	**−1**.**25**	−0.25
HORVU0Hr1G000910	Mannan endo-1,4-β-mannosidase	**1**.**96**	**1**.**00**
HORVU5Hr1G062460	Callose synthase 1	**4**.**48**	**5**.**48**
HORVU3Hr1G097050	Fructan 1-exohydrolase (1-FEH)	**3**.**42**	**3**.**09**
Genes encoding seed storage proteins			
HORVU3Hr1G014090	NAC domain protein 019	**−1**.**33**	−0.33
HORVU1Hr1G077530	bZIP protein BZL2	**−1**.**10**	0.06
HORVU1Hr1G001350	Low molecular weight glutenin	−**2**.**80**	−0.17
HORVU1Hr1G001020	Low molecular weight glutenin	−**1**.**96**	0.18
HORVU1Hr1G064080	High molecular weight glutenin	**−1**.**84**	−0.38
HORVU1Hr1G000700	γ-Gliadin	**−2**.**26**	−0.17
HORVU1Hr1G000680	γ-Gliadin	**−2**.**08**	−0.29
HORVU1Hr1G000640	γ-Gliadin	**−1**.**87**	0.21
HORVU5Hr1G000710	Prolamin, grain softness protein	**−2**.**81**	−0.42
HORVU2Hr1G088760	Caleosin-related family protein	**−1**.**38**	−0.17
HORVU5Hr1G000660	Hordoindoline B1	**−1**.**95**	−0.20
HORVU5Hr1G000680	α-Puroindolin	**−1**.**87**	−0.12
HORVU1Hr1G087820	β-Purothionin	**−1**.**85**	−0.04
HORVU1Hr1G087800	β-Purothionin	**−1**.**56**	0.03
HORVU2Hr1G010030	Arginine decarboxylase 2	**4**.**54**	**3**.**82**
Genes encoding proteins involved in cytokinin signaling			
HORVU4Hr1G001610	His-containing phosphotransfer protein AHP8	**−1**.**17**	−1.03
HORVU4Hr1G001680	His-containing phosphotransfer protein AHP12	−0.93	−0.79
HORVU4Hr1G001720	His-containing phosphotransfer protein AHP13	**−0**.**96**	**−1**.**37**
HORVU4Hr1G001740	His-containing phosphotransfer protein AHP14	**−1**.**43**	**−1**.**79**
HORVU4Hr1G001790	His-containing phosphotransfer protein AHP16	**−1**.**36**	−1.47

Significantly different expression is shown in bold (*P* adj < 0.05. The *P* values were adjusted for multiple testing according to Benjamini–Hochberg FDR correction).

DAF, days after flowering.

Several genes transcriptionally affected by the knock-down of *HvSWEET11b* were associated with the synthesis of endosperm storage proteins. The transcript levels of the genes encoding the transcription factors NAC019 (Gao et al. 2021) and BLZ2 (Onate et al. 1999), which are responsible for the transcriptional activation of genes encoding glutenin and hordeins, respectively, were lower in transgenic versus WT grains (Table 1). As a consequence of the downregulation of these 2 genes, the transcription of various genes encoding low and high molecular weight glutenins and diverse prolamins was negatively affected. Various other genes involved in protein synthesis and degradation were differentially transcribed, as was 1 gene encoding an α- and 2 β-puroindolines.

**Figure 4.**
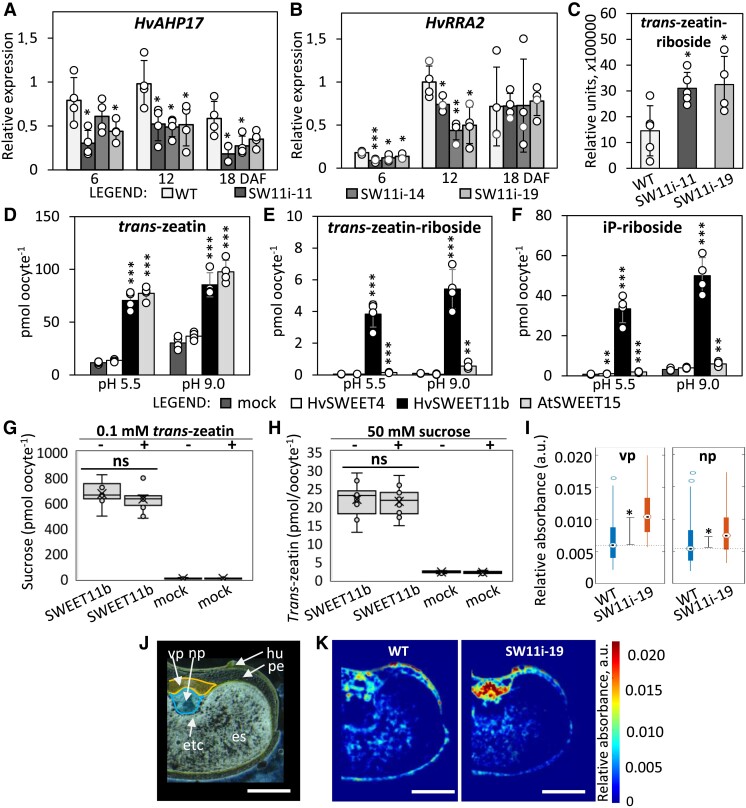
HvSWEET11b acts as a cytokinin transporter. **A** and **B**) Transcript profiles of the AHP *HvAHP17* and RR *HvRRA2* genes in *HvSWEET11b*-repressed grains compared with the WT. Values are means, error bars represent Sd; *n* = 4 biological replicates, each consisting of 4 to 10 caryopses from 3 spikes; *P < 0.05, **P < 0.01, and ***P < 0.001, as determined by 2-tailed Student's *t*-test between WT and transgenic grains. **C**) Accumulated *trans*-zeatin riboside in the transgenic and WT grains at the beginning of the storage phase. Values are means ± Sd, *n* = 5 biological replicates, each consisting of 4 to 10 caryopses from 3 spikes; *P < 0.05 as determined by the 2-tailed Student's *t*-test between WT and transgenic grains. **D**–**F**) When heterologously expressed in *Xenopus* oocytes, HvSWEET11b and AtSWEET15, unlike HvSWEET4, can effectively transport various forms of cytokinin. The assay was done by incubating *SWEET*-expressing oocytes in a mixture of equimolar concentrations of phytohormones for 1 h. Values are means ± Sd; *n* = 4 to 5 biological replicates (individual oocytes) in (D–F). *P < 0.05, **P < 0.01, and ***P < 0.001 as determined by a 2-tailed Student's *t*-test between mock and the oocytes expressing a particular transporter. A competition assay to determine the preference of HvSWEET11b for *trans-*zeatin in the presence of sucrose (**G**) or vice versa (**H**). Nonsignificant (ns) *P* > 0.05, as determined by a 2-tailed Student's *t*-test between the presence and absence of competitor substrate (*n* = 10 to 12 biological replicates). **I**) The accumulation of *trans*-zeatin riboside in the vascular region (vp) and the nucellar projection (np) measured by FTIR microspectroscopy. Data are given as relative units (dot: median, box: interquartile range [IQR], line: 1.5× IQR, asterisk: statistical significance [*P* < 0.05]), as determined using the Mann–Whitney *U*-test. Analysis of 5 biological replicates each consisting of 1,200 ± 400 spectra per selected region. **J**) A grain cross-section shows the tissue structure of the grain. The nucellar projection and vascular bundle with its surrounding pericarp, used for *trans*-zeatin riboside quantifications in (I), are colored blue and yellow, respectively. es, endosperm; hu, husk; np, nucellar projection; pe, pericarp; vp, vascular region with adjacent pericarp. Scale bars = 1 mm. **K**) The distribution of *trans*-zeatin riboside, as visualized by FTIR microspectroscopy in the grains of WT and *HvSWEET11b* knockdown plant. The concentration of *trans*-zeatin riboside is color-coded and given in a.u. Bars = 1 mm. Individual samples are shown as dots in (A–H).

An unexpected consequence of the knockdown of *HvSWEET11b* was the downregulation of several genes associated with cytokinin signaling (Table 1). Cytokinin-mediated signal transduction is governed by a 2-component phosphor-relay system, which operates via histidine kinases, authentic histidine phosphotransferases (AHP), and various other response regulators (RR) (Kieber and Schaller 2018). The barley AHP family comprises 17 members, 14 of which are clustered in a single region of chromosome 4H (Supplemental Fig. S8A). The activity of 10 of the 14 *AHP*s was restricted to the developing grain. These included 4 genes (*HvAHP7*, *HvAHP10*, *HvAHP11*, and *HvAHP15*) that were predominantly transcribed during early development and 6 (*HvAHP6*, *HvAHP8*, *HvAHP12*, *HvAHP14*, *HvAHP16*, and *HvAHP17*) whose peak transcription occurred during grain filling, thereby coinciding with that of *HvSWEET11b* (Supplemental Fig. S8, B and C). RT-qPCR analysis of these 6 AHP genes confirmed that their transcript abundance was reduced in the grains of *HvSWEET11b*-knockdown plants (Fig. 4A; Supplemental Fig. S9; Table 1). Because Type-A RRs transcriptionally respond to cytokinin levels via direct activation by Type-B RRs (Kieber and Schaller 2018), we analyzed their expression in *HvSWEET11b*-repressed grains by RT-qPCR (Fig. 4B; Supplemental Fig. S10). Of the 7 Type-A RR genes in barley (Supplemental Fig. S10A), only *HvRRA2* showed moderate levels of expression in developing grains (Supplemental Fig. S10B), and the transcript abundance of this gene decreased upon *HvSWEET11b* knockdown (Fig. 4B). Altogether, these results point to perturbed cytokinin transport and/or signal transduction.

### The barley protein SWEET11b can transport cytokinins

We performed a heterologous expression experiment using *Xenopus* oocytes to test the potential of SWEETs (specifically HvSWEET11b) to transport any glucosyl ester of abscisic acid (ABA), which appears to be a transported form of ABA (Nambara and Marion-Poll 2005); gibberellic acid 3 (GA_3_) and jasmonic acid-isoleucine (Ja-Ile), the mobile forms of gibberellic acid and jasmonate, respectively; and the cytokinin derivatives *N*6-(Δ2-isopentenyl) adenine riboside (iPR), *trans*-zeatin (tZ), and tZ riboside (tZR), forms of cytokinin that are thought to be transported via the cellular membrane (Osugi et al. 2017; Kieber and Schaller 2018). HvSWEET4 was unable to mediate the transfer of any of the above compounds. HvSWEET11b transported tZ, iPR, and tZR, although not all with the same efficiency level (Fig. 4, D–F and Supplemental Fig. S2, D–F). Neither HvSWEET4 nor HvSWEET11b transported any other phytohormones (Supplemental Fig. S11). AtSWEET15 could also mediate the transport of the 3 forms of cytokinin, but its efficiency was lower than that of HvSWEET11b for both tZR and iPR (Fig. 4, D–F).

Because HvSWEET11b could transport both sucrose and cytokinin, we performed a competition assay to determine whether excess sucrose would inhibit the cytokinin transport activity of this protein and whether the presence of cytokinin would influence the uptake of sucrose. Oocytes expressing *HvSWEET11b* were exposed to 10 mM ^14^C-labeled sucrose, either in the presence or in the absence of 0.1 mM tZ. In parallel, *HvSWEET11b*-expressing oocytes were exposed to 0.1 mM tZ in the presence or in the absence of 50 mM unlabeled sucrose. The sucrose transport activity of HvSWEET11b was not affected by small amounts of tZ, nor did 500-fold higher sucrose concentrations affect tZ uptake (Fig. 4, G and H).

### Suppressing *SWEET11b* alters cytokinin gradients inside the developing grain

Liquid chromatography–mass spectrometry (LC–MS) showed that whole grains sampled at the start of the grain-filling stage from *HvSWEET11b*-repressed plants contained higher levels of tZR than WT grains (Fig. 4C). The use of an improved Fourier transform infrared (FTIR) analysis technique (Guendel et al. 2018, 2021) allowed us to not only quantify tZR in cross sections of transgenic and WT grains, but also to examine its spatial distribution. tZR is the transported form of cytokinin (Kieber and Schaller 2018) and represents the bulk of cytokinin molecules in the developing barley grain (Powell et al. 2013). We acquired the relevant spectra by imaging recrystallized tZR mounted on a high-pressure solid transmission spectroscopy cell (Specac, Orpington, UK). The relative absorbances of tZR in grain cryosections are shown in Supplemental Data Set S3 and Fig. S12. A comparison between the grains of WT and *HvSWEET11b* knockdown plants at the beginning of the filling stage revealed that *HvSWEET11b* repression induced a major difference in the extent to which tZR accumulated (Fig. 4, I–K). tZR accumulation was significantly higher in the maternal vascular bundle, surrounding pericarp, and nucellar projection of transgenic grains compared to the WT (Fig. 4, I and K). Thus, knocking down *HvSWEET11b* induced a build-up of cytokinin, especially in the maternal part of the grain. We therefore profiled the transcription of barley homologs of genes encoding cytokinin transporters, including G-type ABC transporter 14 (*ABCG14*, Ko et al. 2014; Zhang et al. 2014) and purine permease 14 (*PUP14*, Zürcher et al. 2016). We performed this profiling by taking advantage of a pre-existing RNA-seq database containing detailed transcriptome information from 14 barley tissues (Monat et al. 2019). The barley genome harbors 1 homolog of *PUP14* and 3 copies of *ABCG14* genes (Mascher et al. 2017); however, none of these 4 genes appear to be transcribed during grain filling (Supplemental Fig. S13 and Data Set S2). This finding implies that other known cytokinin transporters cannot functionally replace HvSWEET11b in terms of cytokinin transport during grain filling.

### The phenotypes of grains from *HvSWEET11b*-knockdown plants

**Figure 5.**
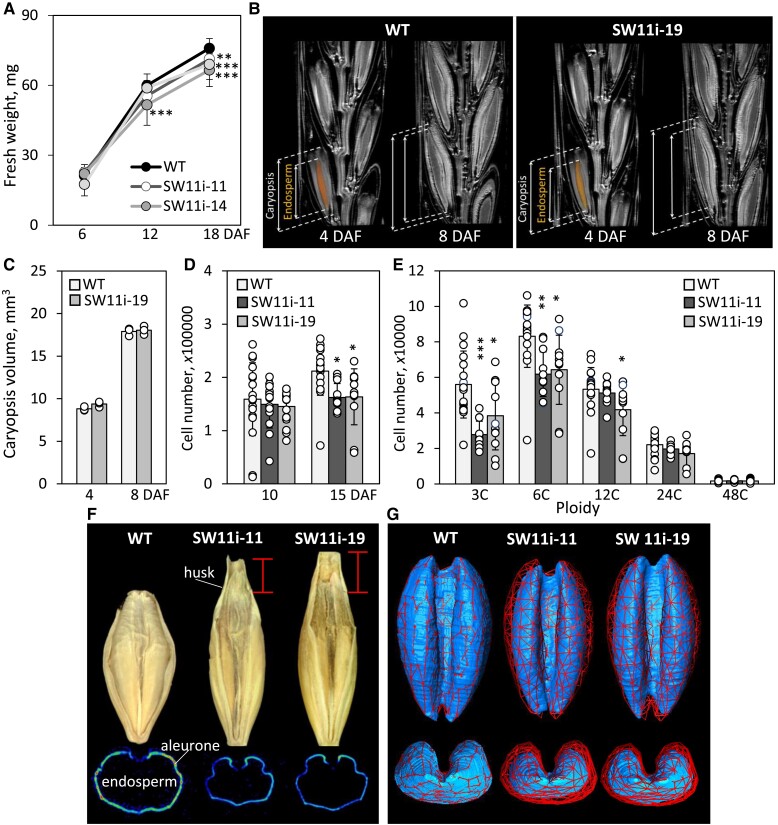
The effect of suppressing *HvSWEET11b* on grain phenotypes. **A**) Transgenic grains accumulate fresh weight less efficiently than WT. **B**) Dynamic MRI visualizes grain expansion in WT and *HvSWEET11b*-repressed plants over time (see Supplemental Movie S1). **C**) The size of developing caryopses at early developmental stages (4 DAF and 8 DAF). **D**) Cell number in the developing endosperm. **E**) Ploidy level of endosperm cells at 15 d after flowering. **F**) The shape of mature WT and *HvSWEET11b*-repressed grains with attached husks (upper panel) and the grain's aleurone layer (lower panel) at virtual cross-sections acquired using MRI. The red lines indicate the greater length of the transgenic grains, resulting from their formation of a longer husk. **G**) The dorsal (upper panel) and posterior (low panel) aspects of the endosperm in developing grains sampled 12 DAF, as acquired using MRI (see also Supplemental Movie S1). The red network defines the outer surface of the WT endosperm (left). The same network is applied for transgenic endosperms to visualize their similar appearance but smaller size than WT. Bars in (E) and (F): 1 mm. Values are means, error bars represent Sd; *n* = 11 to 42 biological replicates (individual caryopses from at least 6 spikes) in (A), *n* = 3 biological replicates (individual caryopses) in (C), *n* = 12 to 18 biological replicates (individual caryopses from at least 4 spikes) in (D, E). *P < 0.05, **P < 0.01, and ***P < 0.001, as determined by 1-way ANOVA with Bonferroni correction test between WT and the corresponding transgenic line. Individual samples are shown as dots in (C–E). DAF, days after flowering.

To define the timepoint at which the size of grains of *HvSWEET11b-*knockdown plants diverged from those of WT plants, we sampled grains during the first 18 d post-anthesis. During the first 6 d, there was no significant difference in fresh weight between transgenic and WT grains, but once grain filling began, the gap between transgenic and WT grains gradually widened (Fig. 5A). An in vivo assay of grain development based on MRI showed that their growth was more or less in step until 8 d after flowering (Fig. 5, B and C; Supplemental Movie S1). Estimates of cell number in the endosperm, as assessed using flow cytometry, showed little difference between the transgenic and WT samples during the early period of grain development. However, by 15 d after flowering, the growth of the transgenic grains was lagging (Fig. 5D). The flow cytometry profiles also revealed a lower number of endopolyploid cells in transgenic endosperm at this stage (Fig. 5E).

Transgenic grains appeared to be thinner and longer than the WT (Fig. 5F). We performed MRI imaging to characterize the structure of the mature grain. WT and transgenic endosperm were of similar shape and appearance, but the size of transgenic endosperm was 60% to 70% that of the WT (Fig. 5, F and G; Supplemental Movie S2). The germination capacity of the transgenic grains was not affected.

**Figure 6.**
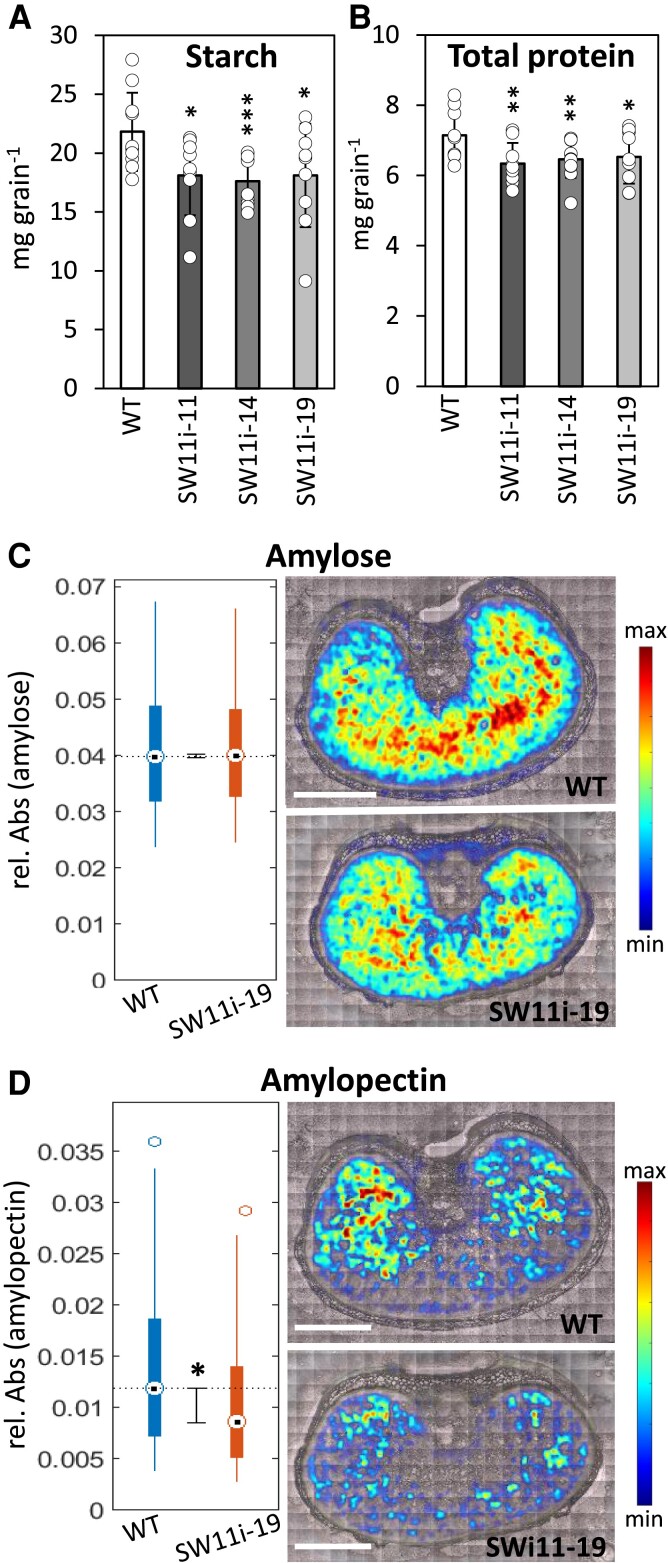
The effect of knocking down *HvSWEET11b* on the accumulation of seed storage compounds. The quantity of starch (**A**) and protein (**B**) present in the mature grains of WT and 3 independent *HvSWEET11b* knockdown transgenic lines. Values are means; error bars represent Sd (*n* = 9 to 11 biological replicates, each consisting of 20 caryopses from individual plants). *P < 0.05, **P < 0.01, and ***P < 0.001 as determined by 2-tailed Student's *t*-test between WT and the corresponding transgenic line. Individual samples are shown as dots. The distribution (right) and the quantity (left) of amylose (**C**) and amylopectin (**D**) present in immature grains set by WT and *HvSWEET11b* knockdown transgenic plants. Data in the plot on the left are given in the form of relative units (dot: median, box: IQR, line: 1.5× IQR, star: statistical significance [*P* < 0.05]), as determined using the Mann–Whitney *U*-test. Analysis of 5 biological replicates each consisting of 45,000 ± 5,000 spectra per selected region (endosperm). Min/max values of the color bar represent the absorbance range, as depicted on the *y* axis in (C) and (D). Bars = 1 mm.

The contents of both starch and protein in mature grains were reduced by *HvSWEET11b* repression (Fig. 6, A and B). An FTIR micro-spectroscopy-based analysis (Gündel et al. 2018) of amylose and amylopectin contents in grains sampled at the filling stage showed that neither the amylose content nor its spatial distribution was altered by the presence of the transgene (Fig. 6C). In contrast, the transgenic endosperm displayed a marked decrease in amylopectin content, especially in its ventral region (Fig. 6D). Overall, we conclude that knocking down *HvSWEET11b* affected cell number and endoreduplication, as well as compromised the accumulation of both starch (especially amylopectin) and storage protein, resulting in the formation of viable but smaller grains.

### Nucleotide diversity of *HvSWEET11b* within barley

**Figure 7.**
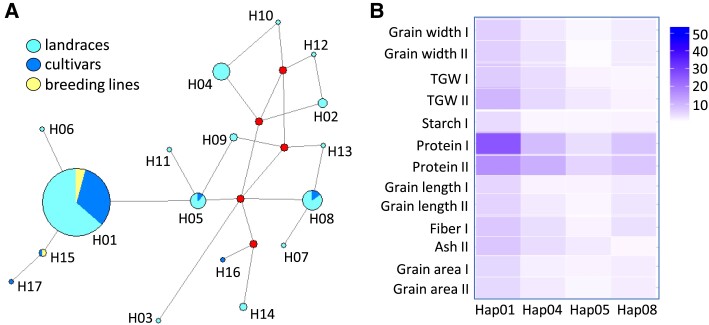
Allelic variation at *HvSWEET11b.***A**) Re-sequencing of the gene in a panel of barley accessions reveals several haplotypes. **B**) The association of grain phenotype with *HvSWEET11b* haplotype. The color-coded scale shows |−log_10_| transformed *P* values.

We carried out a survey of allelic diversity within the *HvSWEET11b* gene using a panel of 314 diverse cultivated spring 2-rowed single-seed descendent barley lines originating from IPK Gene Bank accessions (Pasam et al. 2012, 2014) of wide origin, mainly landraces but also modern cultivars and breeding lines (Supplemental Data Set S4). A *HvSWEET11b* genic region spanning all 5 exons (except for the 26 nucleotides lying at the 3′ end of Exon 5, due to their high CG content) and 4 introns was resequenced. Seventeen haplotypes were identified in the sequenced region (Fig. 7A), with all variant nucleotides lying within intronic or protein-coding sequences (Supplemental Data Set S5). However, no single single nucleotide polymorphism was discovered that altered the amino acid sequence, showing that the HvSWEET11b protein is highly conserved. H01, the overwhelmingly most common haplotype [present in 83% (=263) of the accessions], was represented in landraces, cultivars, and breeding lines. The modern cultivars not carrying H01 carried H05, H08, H15, H16, or H17, while the other 11 haplotypes were only found in landraces. The prevalence of a single haplotype and the lack of nonsynonymous mutations throughout the population indicate that the primary structure of the HvSWEET11b protein is critical for its proper function. To better understand the influence of natural variation in *HvSWEET11b* on agronomic variables, we examined the effects of *HvSWEET11b* haplotypes on various grain-associated traits in a collection of accessions harboring the major haplotypes (H01, H04, H05, and H08). The trial was performed in the field over 2 seasons. Applying a significance threshold of 4.0, we determined that H01 was associated with a higher grain protein content and a larger TGW than the other haplotypes (Fig. 7B).

## Discussion

### HvSWEET11b mediates the movement of both sucrose and cytokinin across the maternal–filial boundary

SWEETs are key components that transfer sugar across the maternal–filial boundary (Chen et al. 2015b; Sosso et al. 2015; Ma et al. 2017; Yang et al. 2018). Five of the 10 barley *SWEET* genes which likely encode SUTs (*HvSWEET11a*, *HvSWEET11b*, *HvSWEET14a*, *HvSWEET14b*, and *HvSWEET15a*) are transcribed in the developing grain (Supplemental Fig. S1A). The extent of the overlap in their temporal and spatial transcription patterns suggests that a degree of functional redundancy exists. However, plants lacking a functional copy of *HvSWEET11b* cannot form viable grains, even though their vegetative growth is not visibly compromised; thus, the *HvSWEET11b* product must play a vital role during the plant's reproductive growth. In rice, it is necessary to simultaneously knock out *OsSWEET11* and *OsSWEET15* to prevent normal grain development (Yang et al. 2018). In barley, even the moderate knockdown of *HvSWEET11b* had phenotypic consequences, namely, altering the allocation of sucrose in the grain during the main filling phase (Fig. 3D), thereby reducing the size of the mature grain (Fig. 3B). When *HvSWEET11b* was heterologously expressed in *Xenopus* oocytes, it not only enabled highly efficient sucrose transport but also facilitated the movement of glucose (Fig. 2). The ability of HvSWEET11b to transport sucrose was shown to be bidirectional and not proton-coupled, as is the case for several other SWEETs (Chen et al. 2010, 2012). Unexpectedly, HvSWEET11b also exhibited the ability to transport cytokinin across the cellular membrane (Fig. 4). While some SWEETs are known to act as transporters of both sugar and gibberellin (Kanno et al. 2016; Morii et al. 2020), we demonstrated that HvSWEET11b can also function as a cytokinin transporter (Fig. 4).

During their development, barley grains accumulate significant quantities of tZ and tZR, while their contents of iP and iPR remain low (Powell et al. 2013). Although the most effectively transported form of cytokinin in *Xenopus* oocytes expressing *HvSWEET11b* was iPR, the predominance of tZR and tZ in developing grains makes it likely that these forms of cytokinin are targeted for transport in planta. A gradient of tZR concentrations was revealed across the grain, with its highest level present in maternal tissue, especially in *HvSWEET11b-*knockdown plants (Fig. 4C), resulting in higher levels of tZR in transgenic grains at the beginning of the storage phase. According to transcriptomic and RT-qPCR analyses, the 6 genes encoding histidine phosphotransferase and the gene encoding the RR HvRRA2 (key cytokinin signaling components) were also downregulated in response to a reduction in *HvSWEET11b* transcript abundance (Fig. 4, A and B; Supplemental Fig. S9).

Whereas some authors have suggested that cytokinin is synthesized early in the developing seed (discussed in Jameson and Song 2016), the transcription of genes encoding the components of cytokinin synthesis in cereals does not correlate well with the sites of cytokinin accumulation during the grain filling stage (Mrízová et al. 2013; Powell et al. 2013; Hluska et al. 2016). Rather, achieving the high levels of tZ and tZR present in barley grains (Powell et al. 2013) must depend on their supply from maternal tissues. Because genes encoding other known cytokinin transporters in barley (such as *ABCG14a*, *ABCG14b*, and *PUP14*) were barely transcribed in the grain during the filling stage (Supplemental Fig. S13), we propose that the protein responsible for mediating the transport of cytokinin across the maternal–filial boundary in barley is HvSWEET11b. The ability of this protein to transport cytokinin was unaffected by sucrose concentrations (Fig. 4, G and H), which correspond to the estimated levels at the unloading zone in barley grains (Guendel et al. 2018). This implies that HvSWEET11b-mediated cytokinin transport can occur under physiological conditions. The observation that the *A. thaliana* SWEET15 protein can also mediate the movement of cytokinins (Fig. 4, D–F) indicated that this dual function of SWEETs is not restricted to barley.

### The relevance of HvSWEET11b to the balance between sink and source

Sugars and cytokinins have been identified as signaling molecules, especially in controlling seed development (Jameson and Song 2016; Wang et al. 2021). Cytokinins interact directly with the plant's cell cycle machinery, as well as affecting grain filling and dormancy (Tuan et al. 2019; Wang et al. 2021); meanwhile, sucrose signaling is intimately linked with the phytohormonal and developmental control of plant growth (Demidchik et al. 2018). Both sugars and cytokinins are brought to the maternal–filial boundary through the maternal plant's vascular system. It therefore might be significant that a single transporter (i.e. HvSWEET11b) is used by barley to enable the transport of both sugars and cytokinins into the developing grain. This dual capacity provides the plant with an efficient means of coordinating grain development and filling. The presence of HvSWEET11b on the maternal side of the boundary during the filling process likely facilitates the release of cytokinin into the endosperm. Increasing cytokinin levels has a positive effect on grain weight (Zalewski et al. 2010, 2014), as demonstrated by the RNAi-silencing of *HvCKX1*, encoding a negative regulator of endogenous cytokinin levels (Holubova et al. 2018) localized to the aleurone layer of endosperm (Mrizova et al. 2013). Cytokinin may support the growth of endosperm, whose cells continue to divide throughout the grain filling period (Olsen 2001, 2020; Chen 2020; Becraft and Gibum 2011). The additional sink created by these tissues increases the grain's overall demand for assimilates, encouraging the HvSWEET11b-mediated release of sugar to the endosperm. Thus, HvSWEET11b activity suggests that cytokinin and sucrose transport function synergistically to control grain development.

The grains of *HvSWEET11b*-knockdown plants formed fewer endosperm cells than those of WT plants, and the mean level of endopolyploidy was lower in these cells (Fig. 5, D and E); to avoid metabolic imbalance, the plant appears to respond by reducing the size of the grains it sets (Fig. 3B). The starvation response to sucrose shortage (Radchuk et al. 2017), including substantial transcriptional reprogramming, activation of starch breakdown, and vacuolar protein degradation, was not evident in transgenic grains. In contrast, an array of morphological and metabolic aberrations is induced when the transfer route of assimilates is interrupted by the repression of either *Jekyll* or *VPE2a-VPE2d* (Radchuk et al. 2006, 2021).

### The role of SWEET11b during the allocation of assimilates in cereal grains

**Figure 8.**
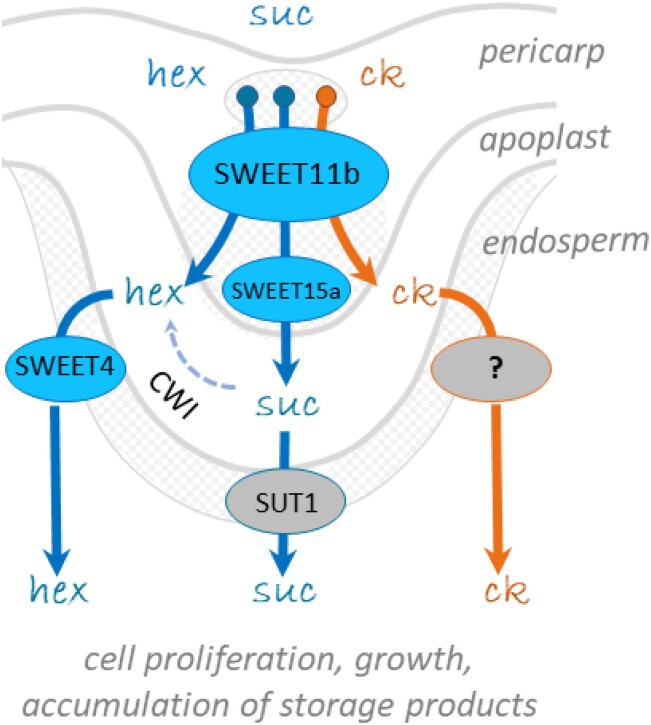
A working model illustrating how the transport of sugars and cytokinins from the mother plant into the developing endosperm of barley grain is coordinated. ck, cytokinin; CWI, cell wall invertase; etc., endosperm transfer cells; hex, hexoses; suc, sucrose.

Cereal species have evolved a variety of structural features that govern the assimilate allocation route (Radchuk and Borisjuk 2014). There are also notable differences between the transcriptional profiles of the barley and rice *SWEET* orthologs. For example, while the peak of transcription of the barley genes *HvSWEET11b*, *HvSWEET15a* (both encoding sugar transporters), and *HvSWEET4* (hexose transporter) coincide during the main filling stage (Supplemental Fig. S1A), the peak time point for *OsSWEET4* expression is earlier during grain development, and that for both *OsSWEET11* and *OsSWEET15* occurs during grain filling (Yang et al. 2018). This difference might, in part, be explained by the finding that barley HvSWEET11b has 2 distinct functions, only one of which is to transport sugar. The implication is that several different pathways through which sugar can flow across the maternal–filial boundary have evolved (Fig. 8). One of these routes relies on the joint action of SWEET and SUT, as suggested by the temporal overlap of *HvSWEET11b* and *HvSWEET15a* transcription (in the nucellar projection) with that of *HvSUT1* (predominantly in endosperm transfer cells; Radchuk et al. 2017). A second pathway mediated by HvSWEET11b involves the release of glucose from the nucellar projection into the apoplastic cavity. The glucose is then imported into endosperm transfer cells by HvSWEET4, a specialized glucose transporter in barley (Fig. 2, A and B) whose orthologs are also present in rice and maize (Sosso et al. 2015). HvSWEET4 activity is favored in an acidic environment, which prevails in the apoplast (Fig. 4F). The cell wall invertases present in the apoplast can cleave sucrose, thereby contributing to the localized accumulation of hexoses during the onset of assimilate storage (Weschke et al. 2003; Wang et al. 2008).

Because the knockdown of *HvSWEET11b* reduced the availability of sucrose in barley endosperm, the protein and starch contents of mature grains set by these plants were lower than those of WT grains (Fig. 6). The starch produced within the transgenic endosperm was particularly deficient in the highly branched polymer amylopectin; the synthesis of this compound requires more energy compared to the linear polymer amylose (Hannah and James 2008). The synthesis of storage proteins is energetically more expensive than that of starch (Vertregt and Penning de Vries 1987). The most common *HvSWEET11b* haplotype represented in a substantial population of 2-rowed spring barleys appears to be associated with both grain size and protein content (Fig. 7). The importance of SWEETs in determining seed size and composition has also been demonstrated in other crop species (Sosso et al. 2015; Wang et al. 2020).

Given the finding that HvSWEET11b mediates the transfer of sucrose, glucose, and cytokinins in *Xenopus* oocytes and that changes in the development of *HvSWEET11b*-repressed grains are associated with perturbed sucrose and cytokinin flow through the maternal tissues toward the endosperm, HvSWEET11b likely plays a multifunctional role in grain development, depending on substrate availability. Because sucrose and cytokinin are transferred by the same transporter, they might exert a synergistic effect on maternal–filial relationships and balance seed growth.

Here we demonstrated that SWEET proteins are multifunctional transporters. In addition to sugars, they may transport phytohormones such as gibberellins (Kanno et al. 2016) and cytokinins (shown here). These findings provide important insights into how plants can transport various compounds using only a limited number of transporters. The dual function of HvSWEET11b in developing grains at the borders of 2 plant generations is an expression of the fact that the roles of sugars and phytohormones in plant development are highly integrated (Waadt et al. 2022; Gautam et al. 2022). The balanced transfer of sugars and plant hormones between generations is likely an important cue in the adaptation of plant growth to an ever-changing environment.

## Materials and methods

### Plant material and growth conditions

WT and the transgenic barley (*H. vulgare* cultivar Golden Promise) plants were grown in controlled environmental conditions under a 16 h light per 18 °C and 8 h dark per 15 °C regime. Supplemental light was set to an intensity of ∼600 *µ*mol quanta m^−2^ s^−1^. The plants were divided into 6 sub-batches. Developing grains from the middle part of the spike were collected separately from each sub-batch and frozen in liquid N_2_. TGW, grain length, and grain width were measured using a Marvin seed analyzer (GTA Sensorik).

### RNA extraction and RT-qPCR

Total RNA was isolated from the samples using Trizol reagent (ThermoFisher Scientific), treated with DNase (Qiagen), and cleaned with a RNeasy kit (Qiagen). The extracted total RNA was reverse-transcribed into single-stranded cDNA using a QuantiTect Reverse Transcription kit (Qiagen). RT-qPCR was performed with a QuantStudio 6 Flex Real-Time PCR system (Applied Biosystems) using PowerUp SYBR Green Master Mix (Applied Biosystems). The relative mRNA levels were determined using the ΔΔCT method and normalized to that of the *H. vulgare actin* gene (HORVU1Hr1G002840). Primers are listed in Supplemental Data Set S6. Experiments were run with 3 to 4 biological replications (taken from independent batches), each with 3 technical repetitions.

### Plasmid construction and plant transformation

The construct targeting *HvSWEET11b* and *HvSWEET15a* by CRISPR/Cas9 was generated using the CasCADE modular vector system. Oligos with complementary target sequences and overhangs for cloning into Bsa I restriction sites were annealed and cloned into Bsa I-linearized vectors pIK1, pIK2, and pIK3, resulting in the vectors pGH757 (*SWEET11b* g1), pGH761 (*SWEET15a* g2), and pGH762 (*SWEET15a* g3). The assembly of all 3 gRNA units in vector pIK61 was achieved by restriction digestion with Esp 3I enzyme and subsequent ligation, giving rise to the vector pGH775. To combine the multiple gRNA fragments from pGH775 with the Cas9 expression unit (pIK83) and an auxiliary unit vector (pIK155) into vector pIK22, Bsa I restriction digestion was performed, followed by ligation. The resulting plasmid was named pGH777. Finally, all expression units of pGH777 were mobilized into binary vector p6-d35S-TE9 (DNA Cloning Service) via Sfi I restriction digestion, resulting in the vector pGH639.

The RNAi construct pSW11bi consisted of the *HvSWEET11b* promoter region (1,700 nucleotides [nt] upstream of the ATG start codon), a sense *HvSWEET11b* fragment (520 nt), an intron of *gibberellic acid oxidase* from potato (*Solanum tuberosum*) (199 nt), an antisense *HvSWEET11b* fragment (520 nt), and the *nopaline synthase* terminator *nosT*. The appropriate DNA fragments were PCR amplified and cloned using specific restriction sites into a modified pAX vector that already contained the potato intron and *nosT*. The primers and restriction sites were as follows: for the *HvSWEET11b* promoter, 5′-CTCGACAGTTAAAGGCCTCTTCAATCTG-3′ (the Stu I restriction site is underlined, as are further restriction sites) and 5′-CACTGCAGCGACGGCTCGG-3′ (Pst I); for the *HvSWEET11b* antisense fragment, 5′-CTGCAGAACTAGTGGAAGAAGACGAC-3′ (Spe I) and 5′-CAGTAGAGCTCGAGCTGGACGCAG-3′ (Xho I); for the *HvSWEET11b* sense fragment, 5′-TGCGGATCCACAGGAAGAAGACGA-3′ (Bam HI) and 5′-CAGTCGACCCCGATCTGGACGC-3′ (Sal I). The pSW11bi cassette was sequenced (LGC Genomics) to confirm the sequences and orientation of the *HvSWEET11b* fragments. The cassette was then introduced into the p6U binary vector (DNA Cloning Service) following digestion with Sfi I. RNAi transgenic and CRISPR/Cas9 mutant barley lines were generated by *Agrobacterium tumefaciens*-mediated transformation of immature embryos of cv. Golden Promise as described (Hensel et al. 2009).

### Phylogenetic analysis

Predicted protein sequences were aligned using ClustalX (Jeanmougin et al. 1998). The phylogenetic trees were constructed using the maximum-likelihood methods in MEGA7 with the following option settings: poisson substitution model, uniform rates, partial deletion for gaps/missing data, 95% site coverage cutoff, strong branch swamp filter and 1,000 bootstrap replications. Sequence alignment is provided in Supplemental Data Set S7.

### Transport assays in *X. laevis* oocytes

The coding DNA sequences of *HvSWEET4*, *HvSWEET11b*, and *HvSWEET15a*, optimized for *X. laevis* expression, were synthesized and cloned into the *Xenopus* expression vector pNB1u at Twist Bioscience. The AtSWEET1 and AtSWEET15 constructs were from Nour-Eldin et al. (2006). Linear DNA templates for in vitro transcription were generated by PCR with pNB1u plasmid-specific primers (Supplemental Data Set S6). Capped cRNA was in vitro synthesized using a mMessage mMachine T7 Kit (Invitrogen), and the cRNA concentration was normalized to 250 ng *µ*L^−1^. Defolliculated *X. laevis* oocytes, Stage V to VI (Ecocyte Bioscience) were injected with 50.6 nl cRNA (or nuclease free water as mock control) using a Drummond NANOJECT II (Drummond Scientific Company) and incubated for 3 d at 16 °C in HEPES-based kulori buffer (90 mM NaCl, 1 mM KCl, 1 mM MgCl_2_, 1 mM CaCl_2_, 10 mM HEPES pH 7.4) supplemented with 100 *µ*g mL^−1^ gentamycin.

^14^C-labeled sugar uptake and export assays were carried out as described (Chen et al. 2012) with the following modifications. For the uptake assay, 3 d after cRNA injection, oocytes were incubated in 150 *µ*L kulori buffer containing 4 *µ*Ci mL^−1^^14^C-glucose (275.0 mCi mmol^−1^, PerkinElmer) or 4 *µ*Ci mL^−1^^14^C-sucrose (435.0 mCi mmol^−1^, PerkinElmer). The final concentration of glucose and sucrose was adjusted to 10 mM by adding unlabeled glucose or sucrose, respectively. After 1 h of incubation (except for the time-course assay), the assay was stopped by adding kulori buffer and the oocytes were washed 4 times in kulori buffer. The oocytes were then transferred individually to scintillation vials and lysed in 100 *µ*L 10% (w/v) SDS by vigorous vortexing. Subsequently, 2.5 mL of EcoScint scintillation fluid (National Diagnostics) was added to each vial (one oocyte/vial), followed by vortexing, and radioactivity was quantified using liquid scintillation counting. Depending on the assay pH, kulori assay buffer pH 5.5 or pH 7.4 was used. For the pH 5.5 assay, oocytes were preincubated for 5 min in MES-based kulori buffer (90 mM NaCl, 1 mM KCl, 1 mM MgCl_2_, 1 mM CaCl_2_, 10 mM MES pH 5.5) prior to assaying.

For the export assay, 3 d after cRNA injection, oocytes were injected with 23 nL of 50 mM glucose (0.05 *µ*Ci mL^−1^^14^C-glucose) or 50 mM sucrose (0.05 *µ*Ci mL^−1^^14^C-sucrose). The oocytes were washed 2 times and incubated in kulori buffer without substrate until the assay was stopped (depending on the time point) by removing the oocytes and washing 3 times. Subsequently, the oocytes were prepared and quantified as described above.

The phytohormone uptake assay was performed as described (Jørgensen et al. 2017) with the following modifications. A mixture of equimolar concentrations (100 *µ*M) of phytohormones (*trans*-zeatin, *trans*-zeatin riboside, isopentenyl adenosine, GA_3_, ABA glucose ester, and jasmonoyl-isoleucine) was used. Three days after cRNA injection, oocytes were preincubated in kulori buffer (pH 5.5) for 5 min, followed by incubation in a mixture of phytohormones (100 *µ*M each) for 1 h. The oocytes were washed 4 times, homogenized in 50% methanol, and stored at −20 °C overnight. Subsequently, the extracts were centrifugated at 15,000 × *g* for 15 min at 4 °C. The supernatant was diluted with water and analyzed by analytical LC coupled to MS (LC–MS/MS) as detailed below.

For assays with mixed substrates, HvSWEET11b-expressing oocytes were incubated in kulori buffer (pH 7.4) containing 10 mM ^14^C-sucrose in the presence or in the absence of 0.1 mM *trans*-zeatin for 15 min. Subsequently, the samples were prepared and radioactivity was quantified using liquid scintillation counting as described above. To test whether HvSWEET11b-mediated *trans*-zeatin could be out-competed by sucrose, HvSWEET11b-expressing oocytes were incubated in kulori buffer (pH 7.4) containing 0.1 mM *trans*-zeatin and 50 mM ^14^C-sucrose in the presence or in the absence of 50 mM unlabeled sucrose for 15 min. D-Sorbitol (50 mM) was used to adjust the osmolarity in the absence of sucrose.

### Phytohormone quantification in oocytes

To measure the phytohormone uptake by the oocytes, samples were diluted 100-fold with deionized water and subjected to analysis by LC–MS/MS and measured in an advance ultra-high-pressure LC (UHPLC) system (Bruker). Separation was achieved on a Kinetex 1.7u XB-C18 column (100 × 2.1 mm, 1.7 *µ*m, 100 Å, Phenomenex, Torrance, CA, USA). Formic acid (0.05%) in water and acetonitrile (supplied with 0.05% formic acid) were employed as mobile Phases A and B, respectively. The elution profile was as follows: 0 to 0.1 min, 5% B; 0.1 to 1.0 min, 5% to 45% B; 1.0 to 3.0 min 45% to 100% B, 3.0 to 3.5 min 100% B, 3.5 to 3.55 min, 100% to 5% B and 3.55 to 4.7 min 5% B. The mobile phase flow rate was 400 *µ*L min^−1^. The column temperature was maintained at 40 °C. The LC was coupled to an EVOQ Elite TripleQuad mass spectrometer (Bruker) equipped with an electrospray ion source. The instrument parameters were optimized by infusion experiments with pure standards. The ion spray voltage was maintained at +5,000 and −3,000 V in positive and negative ion mode, respectively. The cone temperature was set to 350 °C and cone gas to 20 psi. The heated probe temperature was set to 250 °C and probe gas flow to 50 psi. Nebulizing gas was set to 60 psi and collision gas to 1.6 mTorr. Nitrogen was used as the probe and nebulizing gas and argon as the collision gas. Active exhaust was constantly on. Multiple reaction monitoring (MRM) was used to monitor analyte precursor ion → fragment ion transitions. MRM transitions were optimized by direct infusion experiments into the MS source or taken from the literature. Detailed values for mass transitions and references are listed in Supplemental Table S1. Both Q1 and Q3 quadrupoles were maintained at unit resolution. Bruker MS Workstation software (Version 8.2.1, Bruker, Bremen, Germany) was used for data acquisition and processing. Linearity in ionization efficiencies was verified by analyzing dilution series.

### TEVC electrophysiology

The TEVC technique was used to test whether HvSWEET4-mediated glucose transport was H^+^-coupled. The TEVC was performed using automated Roboocyte2 (Multichannel Systems, Reutlingen, Germany). Electrodes were backfilled with a mixture of 3 M KCl and 1.5 M potassium acetate. Oocytes expressing HvSWEET4, AtSUC1 (positive control), and mock (water-injected) oocytes were clamped at −60 mV membrane potential and currents were measured under continuous perfusion of MES-based ekulori buffer (2 mM LaCl_3_, 90 mM NaCl, 1 mM KCl, 1 mM MgCl_2_, 1 mM CaCl_2_, 10 mM MES pH 5.5) for 60 s. Substrate (10 mM glucose for HvSWEET4 and mock, and 0.1 mM sucrose for AtSUC1 oocytes) was then added for 110 s, and the sugar was removed.

### In situ hybridization

Barley caryopses were fixed using FAA fixative (50% EtOH, 5% acetic acid, 10% formaldehyde) overnight in 4 °C, washed 2 times in 50% EtOH, dehydrated, and infiltrated with limonene, followed by Paraplast (Paraplast Plus, Leica) at 60 °C. The samples were embedded and cut into 12 *µ*m thick transverse sections using a Histocore Autocut (Leica). Probe fragments were PCR-amplified using gene-specific primers (Supplemental Data Set S6) with preintegrated T3 or T7 promoter sequences. The probes were labeled with digoxigenin during in vitro transcription using a DIG RNA Labelling Kit (Roche) to produce sense and antisense riboprobes. The tissue samples were treated with proteinase K and hybridized with sense and antisense probes overnight at 55 °C. The digoxigenin incorporated into the probes was detected using an Anti-Digoxigenin-AP, Fab fragments (Roche) and a Vector Blue Alkaline Phosphatase (Blue AP) Substrate Kit (Vector Laboratories), and the sections were examined under a light microscope (Apotome Zeiss).

### Transcriptome analysis

RNA, isolated as described above from whole grains of WT and SW11i-19 transgenic plants at 6 and 12 DAF, was used for library preparation with a SENSE mRNA-Seq Library Prep Kit (Lexogen). The libraries were sequenced on the HiSeq 2500 (Illumina) platform to generate 100-base pair paired-end reads. On average, 17 million reads with mean Q30 of 93.6% and Phred quality score (*Q* score) of 37.4 were generated per sample. Raw reads were trimmed with sickle software (https://github.com/najoshi/sickle) and subjected to quality control (QC) using FastQC. The reads were then aligned to the reference cDNA of *H. vulgare* Morex v2 using kallisto (Bray et al. 2016). The reference cDNA and annotation files were downloaded from Ensembl plants, release 47. Data normalization, filtering, and differential gene expression analysis were done using the R statistical software and limma-voom pipeline (Ritchie et al. 2015). Differential expression thresholds were set at *P* adjusted value <0.05. The *P* values were adjusted for multiple testing according to the Benjamini–Hochberg FDR correction.

### Flow cytometric analysis

Nuclei were isolated from grains with detached embryos as described (Dolezel et al. 2007) using nuclear isolation buffer (Galbraith et al. 1983) supplemented with 50 *μ*g mL^−1^ propidium iodide and 50 *μ*g mL^−1^ DNase-free RNase and counted on a CyFlow Space flow cytometer (Sysmex Europe) using the “Absolute Cell Counting” function. The nuclei were separated from cell debris by gating the corresponding populations in a side scatter/fluorescence dot plot. The proportion of nuclei per endopolyploidy level was calculated based on a log scale histogram displaying the fluorescence intensity of the nuclei stained by propidium iodide.

### MRI

Noninvasive grain analysis was performed using an Avance III HD 400 MHz NMR-spectrometer (Bruker Biospin). Structural ^1^H imaging of living spikes was performed using a 3D spin echo sequence. The repetition time (TR) of these experiments was 700 ms, echo time (TE) = 6.4 ms, field of view (FOV) = 15 × 7 × 7 mm, and 250 × 116 × 116 spatial points were acquired, resulting in a resolution of 60 × 60 × 60 μm, with a total measurement time of Ttot = 2 h 36 min 59 s. An image of single grains was also acquired using a 3D spin echo sequence, but with the following parameters: TR = 800 ms, TE = 6.9 ms, FOV = 15 × 9 × 9 mm, matrix size 250 × 150 × 150, resulting in a spatial resolution of 60 × 60 × 60 *µ*m. Acquiring 4 images for averaging led to a total measurement time of Ttot = 20 h 0 min 0 s.

Dynamic ^13^C/^1^H imaging experiments were performed by detecting ^13^C directly using an adjusted chemical shift imaging protocol applied in spin-echo mode as described (Melkus et al. 2011) in combination with cryogenically cooled double-resonant ^1^H/^13^C probe (Bruker). The SNR was improved by Nuclear Overhauser Effect, an MLEV-pulse scheme was applied for 2,000 ms on the ^1^H channel. A 2-mm slice was excited with “calculated” pulses (excitation: pulse duration [tp] = 168 *µ*s, bandwidth [BW] = 25 kHz; refocusing: tp = 227 *µ*s, BW = 15 kHz). During acquisition, the MLEV scheme was also used for broadband ^1^H signal decoupling. The TR of the sequence was 2.1 s, TE was 2.1 ms, number of averages 64. Five hundred and twelve spectral points were acquired at a receiver BW of 30 kHz. The experiments were performed with an isotropic FOV of 4.4 mm using an acquisition-weighted k-space sampling scheme. The in-plane resolution was 660 × 660 *µ*m and the duration of the experiment 58 min 37 s. As a reference image, a standard 2D spin-echo sequence was used with the following parameters: TR = 1,000 ms, TE = 6.9 ms, matrix size 150 × 150, in-plane resolution 29 *µ*m isotropic, slice thickness 500 *µ*m. Processing of data from the NMR experiments was performed using MATLAB (MathWorks). Segmentation and grain modeling were performed using AMIRA software (FEI Visualization Sciences Group).

### FTIR imaging and data processing

Samples were frozen in liquid nitrogen and embedded in Tissue-Tek cryomolds using Tissue-Tek O.C.T. (Sakura Finetek) at −20 °C. Embedded tissues were cross-sectioned (16 *µ*m) with a cryotome CryoStar NX7 (ThermoFisher Scientific) and transferred onto molecular machines and industries membrane slides. Tissue sections were lyophilized and stored in the dark at room temperature until analysis. These slides were also used for internal standardization as described below.

Imaging was performed using a Hyperion 3000 FTIR microscope (Bruker Optics) coupled to a Tensor 27 FTIR spectrometer (Bruker Optics) with an internal midinfrared source. The focal plane array detector (64 × 64 pixel) was used in transmission mode. The imaging system was continuously purged with dry air. FTIR images were recorded in the spectral range of 3,900 to 800 cm^−1^ at a spatial resolution of 11 *µ*m and a spectral resolution of 6 cm^−1^ using 3.5× (15× for high detail images; 5.5 *µ*m digital resolution with 2 × 2 pixels binning) infrared magnification objectives (Bruker Optics). Each spectrum comprised 64 coadded scans. A reference of a single focal plane array window of the empty light path was acquired before image acquisition and automatically subtracted from the recorded image using OPUS software (Bruker Optics). Atmospheric absorptions of water vapor and CO_2_ were corrected by OPUS during image acquisition. OPUS files were imported into MATLAB (MathWorks) as ENVI files using the multiband-read function or the irootlab toolbox (Trevisan et al. 2013). Spectral features such as carbohydrates and sucrose fingerprints, along with baseline features, were extracted using an extended multiplicative signal correction model adopted into an inhouse developed analytical MATLAB routine for statistical and quantitative spectral feature analysis as described by Guendel et al. (2018, 2021). The spectral library used for modeling includes the major plant hormones and some derivatives, a selection of amino acids, secondary metabolites, carbohydrates, lipids and proteins, atmospheric spectra, baseline scatter, and the internal standard (Supplemental Data Set S3).

tZR was validated using a methanol stock solution and applied in droplets by micro syringe in 4 stages up to 0.4, 4, 10, and 20 *µ*L. The tissue section was imaged after each standard application and modeled based on unaltered tissue sections. For each image, the total amount each compound was plotted against the added tZR standard and fitted by linear regression. Fits were evaluated according to performance parameters such as RPD (ratio of performance of deviation) and the signal-to-noise ratio of signal difference from untreated tissue over predictive error.

### Metabolome profiling

For untargeted analysis of central metabolites, freeze-dried samples were extracted as described (Radchuk et al. 2021), followed by ion chromatography (IC) using the Dionex ICS-5000+ HPIC-system (Thermo Scientific) coupled to a QExactive-Plus hybrid quadrupole-orbitrap mass spectrometer (Thermo Scientific). The detailed chromatographic and MS conditions are described in Supplemental Table S2. The samples were randomized and analyzed in full MS mode. Data-dependent MS–MS analysis for compound identification was performed in the pooled probe. The batch data were processed using the untargeted metabolomics workflow of Compound Discoverer 3.0 software (Thermo Scientific). The alignment of retention times was performed using an adaptive curve model with maximum shift of 0.5 and 5 ppm mass tolerance. Feature detection was set at minimum peak intensity of 10,000, signal-to-noise threshold of 3 and 3 ppm mass tolerance. Peak area drift correction was performed using the cubic spline algorithm based on the repeated pooled probe as a QC. Compounds with a maximum relative standard deviation of 35% of the QC area were selected for quantification. Additionally, the overall stability of sample recovery was monitored using levulinic acid as an internal standard. The compounds were identified using an inhouse spectral library and a mass list, as well as the public spectral database mzCloud and mass databases ChemSpider, KEGG, and Metabolika. The *P* values of the group ratio were calculated by ANOVA and Tukey–HCD post hoc analysis. In some cases, compounds were manually reintegrated using TraceFinder4.1 software (Thermo Scientific), followed by peak area correction using the MetaboDrift Excel-software component. Untargeted profiling of amino acids and some other cationic metabolites was performed using the vanquish focused UHPLC system (Thermo Scientific) coupled to the same mass spectrometer. The batch processing and compound identification workflow was essentially the same as that described for IC–MS-based untargeted profiling.

### Analysis of cytokinin levels in caryopses

To analyze the concentrations of cytokinins in caryopses, freeze-dried samples were extracted and purified following Šimura et al. (2018). The analysis was performed using LC/MS Dionex UPLC-system (Thermo Scientific) coupled to a QExactive-Plus hybrid quadrupole-orbitrap mass spectrometer in targeted single ion monitoring mode as described in Supplemental Table S2.

### Measurement of pH in the apoplastic space of developing barley grains

Needle-type NHT-HP5 microsensors with a 140-*µ*m diameter connected to a pH micro device (PreSens, Precision Sensing GmbH) were manually inserted into the liquidus phase of the endosperm cavity to measure pH value.

### Vitality staining of pollen

Pollen grains were fixed and stained essentially as described (Peterson et al. 2010).

### Near infrared spectroscopy

Grain composition was measured in diffuse reflectance using a near infrared spectroscope (Bruker MPA) in powdered samples according to the supplier's protocol (B-FING-M, Bruker) using OPUS measurement software with integrated calibration.

### SNP detection, haplotype analysis, and population genetic analysis

The nucleotide diversity of *HvSWEET11b* was analyzed in a collection of 314 barley accessions comprising only 2-rowed spring modern and older cultivars and an enlarged number of landraces to capture high phenotypic and genotypic diversity (Supplemental Data Set S4). The collection was grown for 2 yr (2017 and 2018) in the fields of IPK campus in 2 replicates employing 3 m^2^ plots for each accession organized in a randomized block design. The yield-relevant parameters (TGW, grain length, grain width, grain area, starch, protein, fiber, and ash contents) were phenotyped and evaluated.

Total genomic DNA was extracted from the samples and PCR amplified with *HvSWEET11b*-specific primers (Supplemental Data Set S6) as described (Radchuk et al. 2019). The amplicons were cleaned and sequenced (LGC Genomics, Germany). The sequences were processed with AB DNA Sequencing Analysis Software 5.2. Sequence alignments were generated with ClustalW, and the allelic haplotypes were defined by DNASP 6.12.03 (Librado and Rozas 2009). All singletons were subsequently confirmed via 3 additional independent amplifications and sequencing. A median-joining network (Bandelt et al. 1999) was generated for *HvSWEET11b* haplotypes using the Network 5.0.0.2 program (Fluxus Technology Ltd). ANOVA was used to associate grain traits and haplotypes. Minor haplotypes were not considered for this analysis. Analysis was performed in R version 4.0.5 (http://www.R-project.org/).

### Calculations and statistics

Mathematical calculations and statistical analyses were performed using Excel 2010 (Microsoft Corp., Redmond, WA, USA) and MATLAB software (version R2019b, http://www.mathworks.com). The significance of differences between mean abundances was tested as indicated in the respective figure legends. The outcomes of statistical tests are given in Supplemental Data Set S9.

### Accession numbers

The RNAseq row and processed data were submitted to NCBI BioProject (ID: 852376). The accession numbers of the genes, analyzed in the paper, are listed in the Supplemental Data Set S8.

## Supplementary Material

koad055_Supplementary_Data

## Supplemental data

The following materials are available in the online version of this article.

**Supplemental Figure S1**. Tissue-specific expression of SWEETs.

**Supplemental Figure S2**. Confirmation of the transporting capabilities of the selected SWEET proteins of barley and Arabidopsis using independent batches of *Xenopus* oocytes.

**Supplemental Figure S3**. Two-electrode voltage-clamp measurements on HvSWEET4-mediated glucose uptake in *Xenopus* oocytes.

**Supplemental Figure S4**. Production of the double mutated *HvSWEET11b* and *HvSWEET15a* barley plants using CRISPR/Cas9 technology and the phenotype of the homozygous *HvSWEET11b* mutated plants.

**Supplemental Figure S5**. Molecular analysis of selected primary transgenic lines.

**Supplemental Figure S6**. Transcript profiles of *HvSWEET11a* and *HvSWEET15a*, and a heatmap showing differences in metabolite abundances in *HvSWEET11b*-repressed grains compared with WT at 12 DAF.

**Supplemental Figure S8**. The *AHP* gene family in barley.

**Supplemental Figure S9**. Authentic histidine phosphotransferase genes *HvAHP6, HvAHP8, HvAHP12*, and *HvAHP14* are downregulated in transgenic *HvSWEET11b*-repressed developing grains compared to the WT, as analyzed by RT-qPCR.

**Supplemental Figure S10**. The *response regulator A* (*RRA*) gene family in barley.

**Supplemental Figure S11**. Functional analysis of the selected SWEET proteins on the ability to transport the mobile forms of jasmonic and gibberellic acids in the *Xenopus* oocytes.

**Supplemental Figure S12**. Validation of the *trans*-zeatin riboside measurements by FTIR micro-spectroscopy.

**Supplemental Figure S13**. The expression of barley orthologs of G-type ABC transporter *ABCG14* and purine permease *PUP14* in comparison with *SWEET14b* in 14 different barley tissues as revealed by RNAseq.

**Supplemental Table S1**. MRM transitions for LC–MS/MS analysis.

**Supplemental Table S2**. Chromatographic and mass spectrometry conditions of LC–MS metabolite analyses.

**Supplemental Data Set S1**. Untargeted metabolite analysis of developing grains in response to suppression of *HvSWEET11b*.

**Supplemental Data Set S2**. RNA-seq analysis of WT and *HvSWEET11b*-repressed caryopses at 6 and 12 d after flowering.

**Supplemental Data Set S3**. Overview of spectral library components and derived image variables.

**Supplemental Data Set S4**. List of barley accessions used in *HvSWEET11b* gene diversity study.

**Supplemental Data Set S5**. Nucleotide sequences of the *HvSWEET11b* haplotypes.

**Supplemental Data Set S6**. List of primers used in the work.

**Supplemental Data Set S7**. Sequence alignment of SWEET proteins.

**Supplemental Data Set S8**. Accession numbers of sequences from barley and rice used in the study.

**Supplemental Data Set S9**. Original data and statistical test results.
