# Supplementary material for: SWEET11b transports both sugar and cytokinin in developing barley grains
Source: Plant Cell. 2023 Mar 1;35(6):2186–207. doi: 10.1093/plcell/koad055 (PMC10226576; doi:10.1093/plcell/koad055)
Supplement: koad055_Supplementary_Data [file koad055_supplementary_data.zip › TPC2022RA00967DR1_Supplemental_Data.pdf]

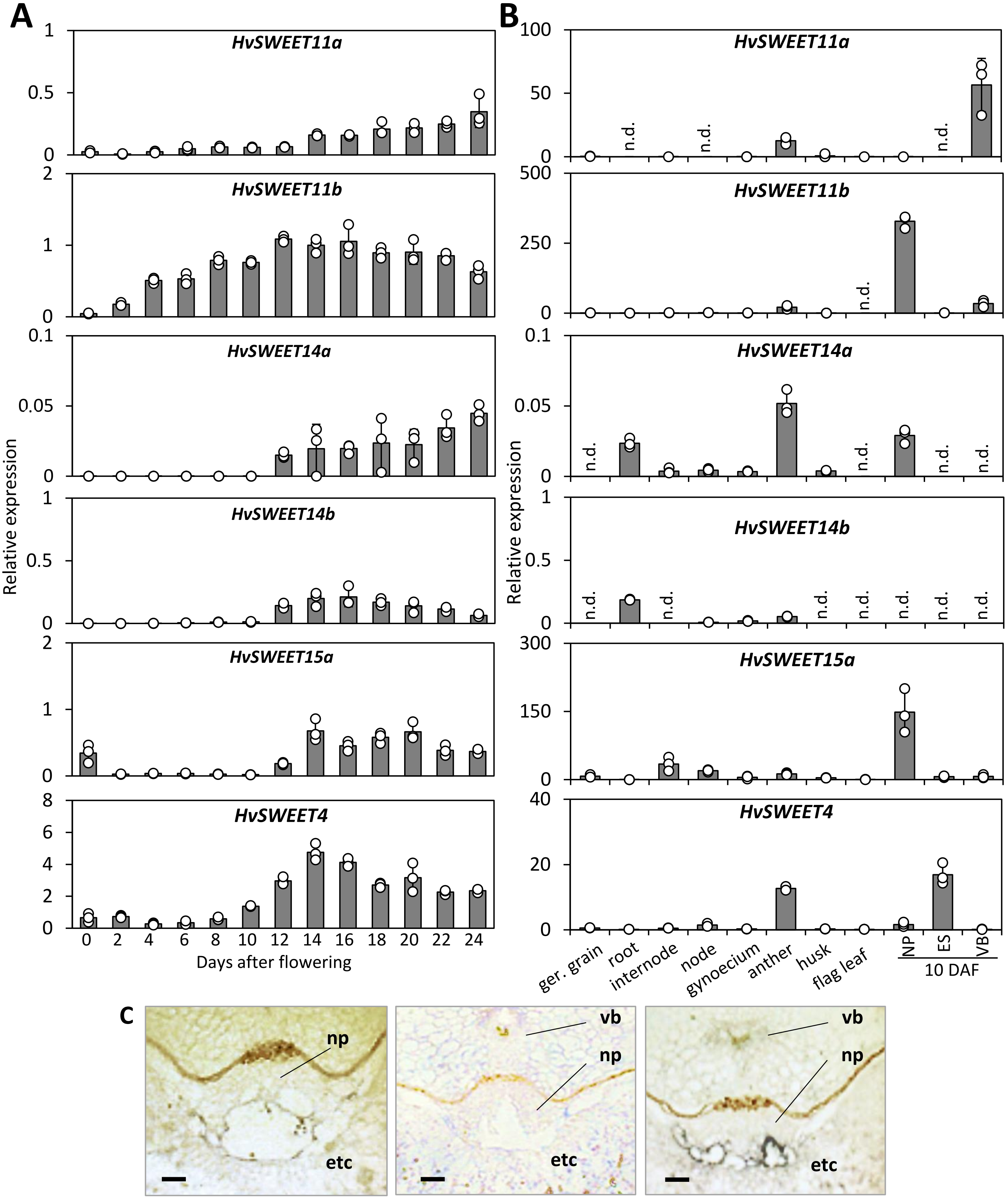

Supplemental Figure S1. **Tissue-specific expression of SWEETs (Supports Fig. 1).**
Transcript abundances of genes encoding the potential sucrose-transporters HvSWEET11-HvSWEET15 and the hexose-transporter HvSWEET4 in developing grains (**A**) and other tissues (**B**) of barley, as assessed by qRT-PCR. (**C**) Control experiment for mRNA *in situ* hybridizations: sense probes for *HvSWEET4* (left), *HvSWEET11b* (middle) and *HvSWEET15a* (right). Scale bars = 100 µm. Data are shown as the mean ± standard deviation (n = 4 biological replicates, each consisting of 4-10 caryopses from three spikes in (**A**), = 3 biological replicates each derived from microdissection of three caryopses in (**B**). Abbreviations: es, endosperm; etc, endosperm transfer cells; n.d., the expression not detected; np, nucellar projection; vb, vascular bundle.

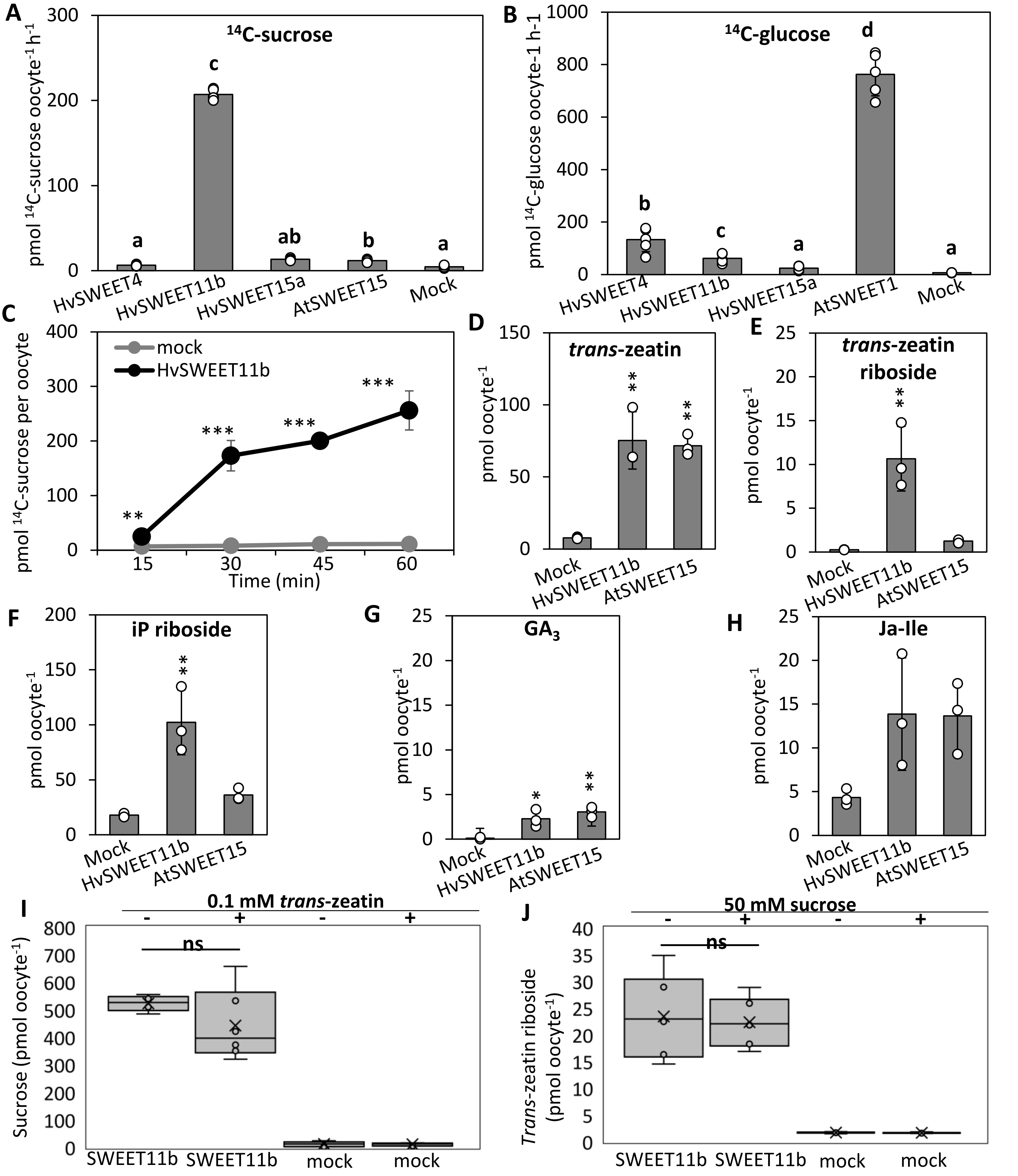

Supplemental Figure S2. Confirmation of the transporting capabilities of the selected SWEET proteins of barley and Arabidopsis using independent batches of *Xenopus* oocytes. (Supports Figs. 2 and 4).

(**A** and **B**) The ability of the barley SWEETs to mediate the transport of sucrose (**A**) and glucose (**B**). (**C**) Uptake of ^{14}C-sucrose mediated by HvSWEET11b. (**D**-**H**) The ability of HvSWEET11b and AtSWEET15 to transport *trans*-zeatin (**D**), trans-zeatin riboside (**E**), iP riboside (**F**), gibberellic (GA_3, **G**) and jasmonic (Ja-Ile, **H**) acids in *Xenopus* oocytes. (**I** and **J**) A competition assay to determine the preference of HvSWEET11b for the trans-zeatin in the presence of sucrose (**I**) or vice versa (**J**).

All values are means ± standard deviation (SD). Different letters in **A** and **B** indicate significant differences at $P < 0.001$ determined by one-way ANOVA with the Bonferroni correction test (n = 5 biological replicates consisting of individual oocytes). n = 4-5 in **C**; = 3 in **D**-**H**; = 5-6 in **I** and **J** *$P < 0.05$, **$P < 0.01$ and ***$P < 0.001$ as determined by two-tailed Student's *t*-test between mock oocytes and oocytes expressing corresponding protein. Individual samples are shown as dots in (**A**, **B**, **D-J**). ns, non-significant.

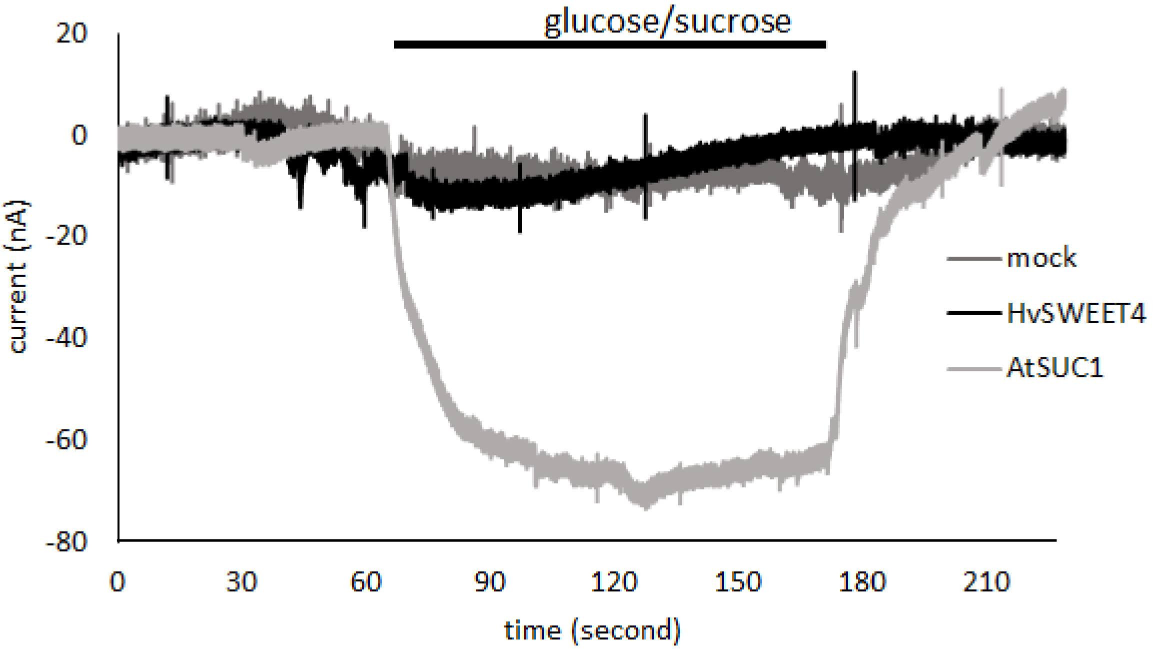

Supplemental Figure S3. Two-electrode voltage-clamp measurements on HvSWEET4-mediated glucose uptake in *Xenopus* oocytes. (Supports Fig. 2). Oocytes expressing HvSWEET4, AtSUC1 (positive control), and mock (water-injected) oocytes were clamped at -60 mV membrane potential and currents were measured under continuous perfusion of Kulori buffer (pH 5.5) for 60 s before the substrate (10 mM glucose for HvSWEET4- and 0.1 mM sucrose for AtSUC1-expressing oocytes) was added for 110 s and then the sugar was removed. The data are shown from a single representative oocyte.

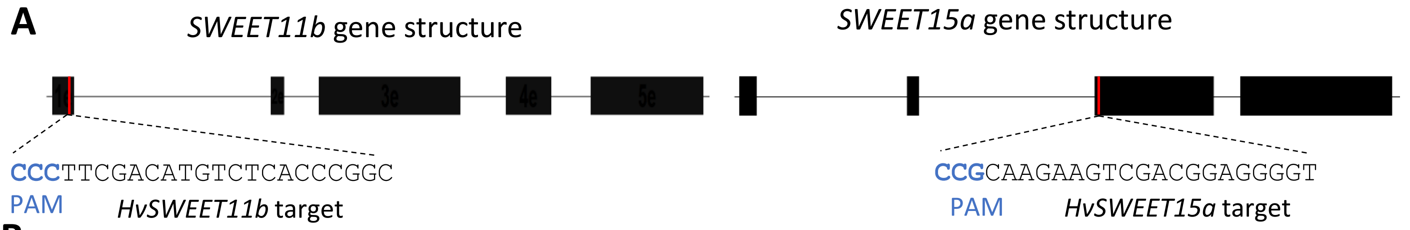

B

Plant nr.	Target sequence in SWEET11b	Indel length	Zygo-sity	Target sequence in SWEET15a	Zygo-sity	Indel length	Fertility
WT	GGCCCCTTC GACATGTCTCAC		–	ACCGCAA GAAGTCGACGGAGG	–		
1-1	GGCCCCTTCAGACATGTCTCAC	+1	**homo**	ACCGCAAAGAAGTCGACGGAGG	homo	+1	**sterile**
1-3	GGCCCCTTCAGACATGTCTCAC	+1	**homo**	ACCGCAAAGAAGTCGACGGAGG	homo	+1	**sterile**
1-6	GGCCCCTTCtGACATGTCTCAC	+1	hetero	ACCGCAAAGAAGTCGACGGAGG	homo	+1	fertile
1-15	GGCCCCTTCAGACATGTCTCAC	+1	**homo**	ACCGCAAAGAAGTCGACGGAGG	homo	+1	**sterile**
3-2	GGC------- -----GTCTCAC	-11	**homo**	ACCGCAA GAAGtcgacggAGG	hetero	-7	**sterile**
3-5	GGCcccttc gacatGTCTCAC	-11	hetero	ACCGCAA GAAGtcgacggAGG	hetero	-7	half-fertile
3-10	GGCcccttc gacatGTCTCAC	-11	hetero	ACCGCAA GAAGtcgacggAGG	hetero	-7	half-fertile
3-12	GGCcccttc gacatGTCTCAC	-11	hetero	ACCGCAA GAAGtcgacggAGG	hetero	-7	half-fertile
31-1	GGCCCCTTCAGACATGTCTCAC	+1	**homo**	ACCGCAA GAAGTCGACGGAGG	WT	no	**sterile**
31-2	GGCCCCTTCaGACATGTCTCAC	+1	hetero	ACCGCAA GAAGTCGACGGAGG	WT	no	half-fertile
31-4	GGCCCCTTC GACATGTCTCAC	no	WT	ACcgcaa gaagtcGACGGAGG	hetero	-11	fertile
31-9	GGCCCCTTCaGACATGTCTCAC	+1	hetero	AC----- ------GACGGAGG	**homo**	-11	half-fertile
31-12	GGCCCCTTCAGACATGTCTCAC	+1	**homo**	ACcgcaa gaagtcGACGGAGG	hetero	-11	**sterile**

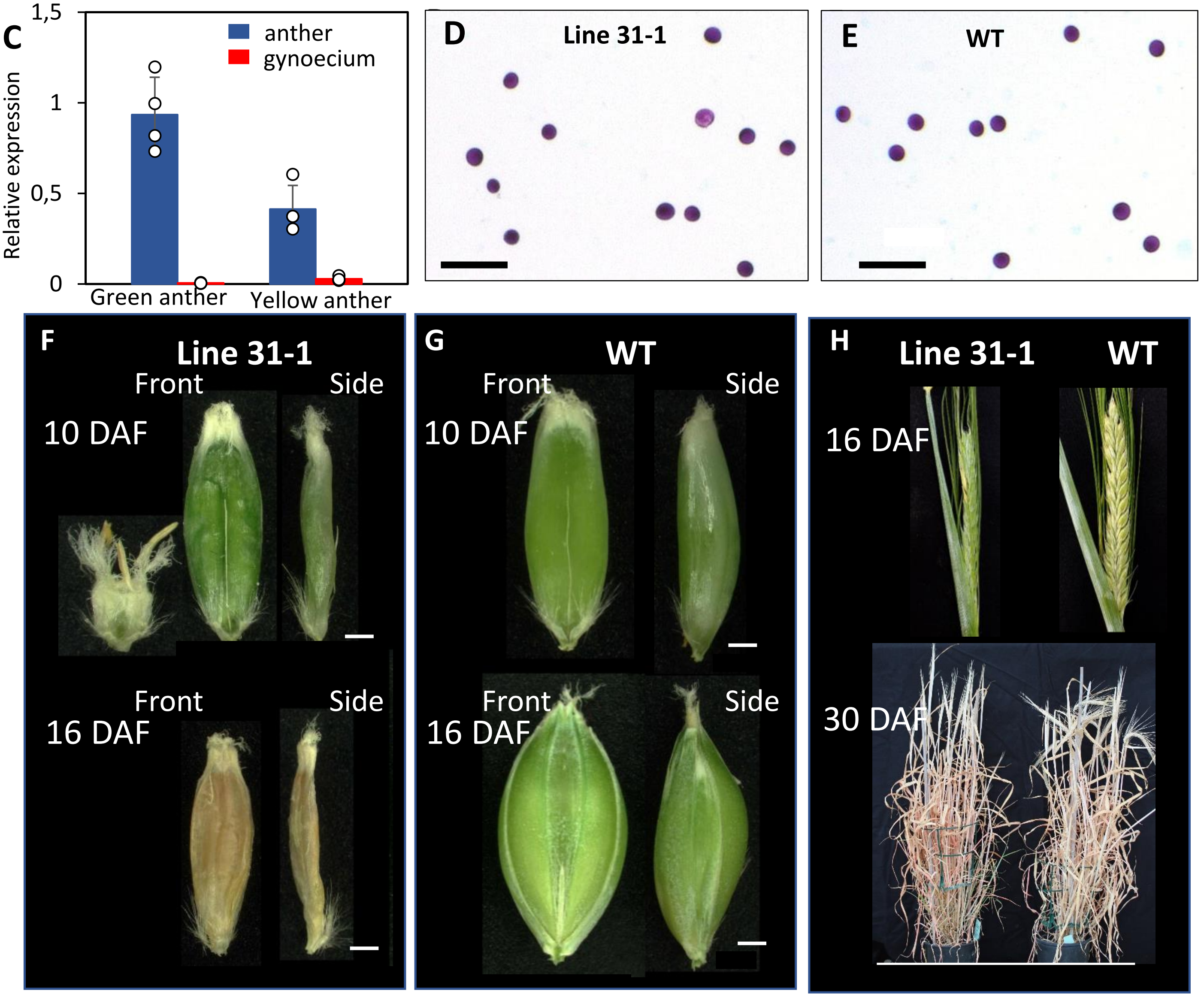

Supplemental Figure S4. Production of double mutated *HvSWEET11b* and *HvSWEET15a* barley plants using CRISPR/Cas9 technology and the phenotypes of homozygous *Hvsweet11b* mutant plants. (Supports Fig. 3).
(**A**) *HvSWEET11b* (left) and *HvSWEET15a* gene models (right) with sequences (shown underneath) targeted by guide RNAs. Exons are shown as black bars, introns as lines. The positions of target sequences in the genic models are labeled in red, protospacer adjacent motif (PAM) is labeled in blue.
(**B**) The CRISPR/Cas9-targeted sequence regions of *HvSWEET11b* and *HvSWEET15a* genes in T_2 transgenic plants. Dashes indicate deleted bases. Nucleotide insertions/deletions are shown in red and as +/-, correspondingly. Small letters indicate the heterozygotic state of the indel.
(**C**) The expression of *HvSWEET11b* in developing anthers and gynoecia. Data are shown as the mean ± standard deviation (n = 4 biological replicates, each consisting of 20-25 flowers from at least 4 different spikes).
(**D**, **E**) Vitality staining of pollen of the mutant line 31-1 (**D**), which is homozygous for *HvSWEET11b* mutation and does not have mutations in *HvSWEET15a*, and wild-type (WT) (**E**) using a protocol by Peterson et al. (2010).
(**F**) The caryopses of *HvSWEET11b*-mutated homozygous line 31-1, front and side views at 10 and 16 days after flowering (DAF). While some caryopses carrying a single mutation in *HvSWEET11b* in a homozygous state died soon after flowering, the vast majority (~90%) reached the early filling stage and died after that.
(**G**) WT caryopses: front and side views at 10 DAF (top) and 16 DAF after flowering.
(**H**) Compared with WT, the phenotypes of the mature homozygous HvSWEET11b-mutated line 31-1. The mutated plant produced spikes that did not contain viable seeds.
Bars: = 200 µm in (**D, E**), = 1 mm in (**F, G)**, = 1 cm in (**H**, upper panel), 50 cm in (**H**, lower panel).

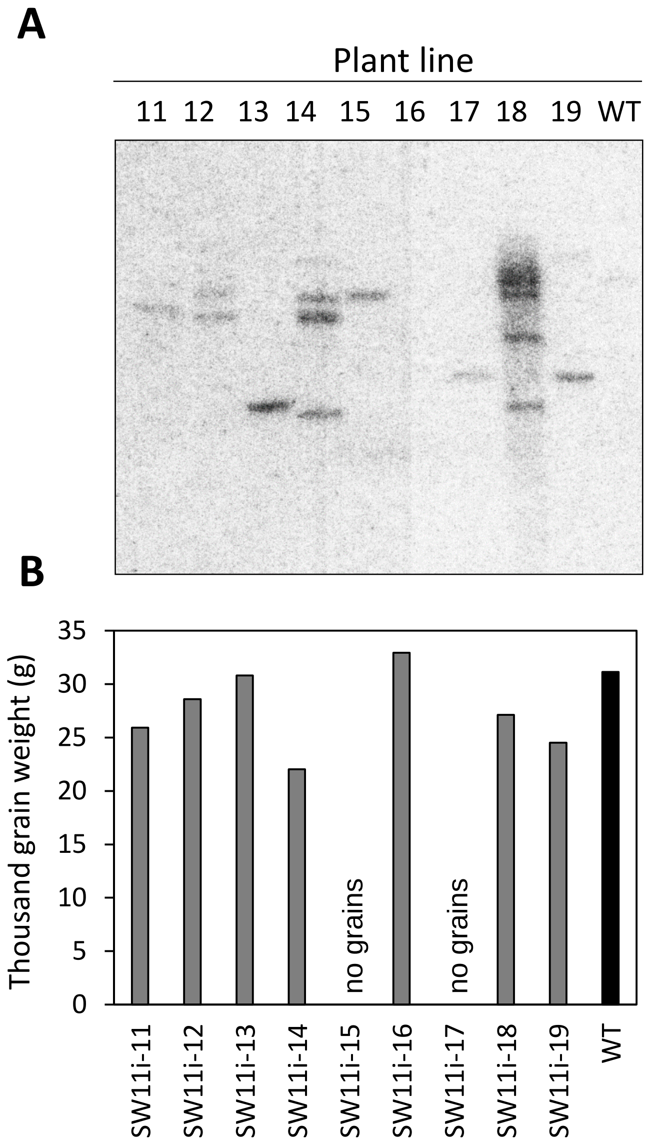

Supplemental Figure S5. **Molecular analysis of selected primary transgenic lines. (Supports Fig. 3).**

(**A**) DNA gel blot hybridization of primary transgenic lines with a fragment of the *hygromycin phosphotransferase* gene as a probe.

(**B**) Thousand grain weight of T_1 heterozygous grains. Because the grains were collected from single T_0 plants, no statistical analysis was possible. The data were used to select transgenic lines to produce the homozygous lines for detailed analyses.

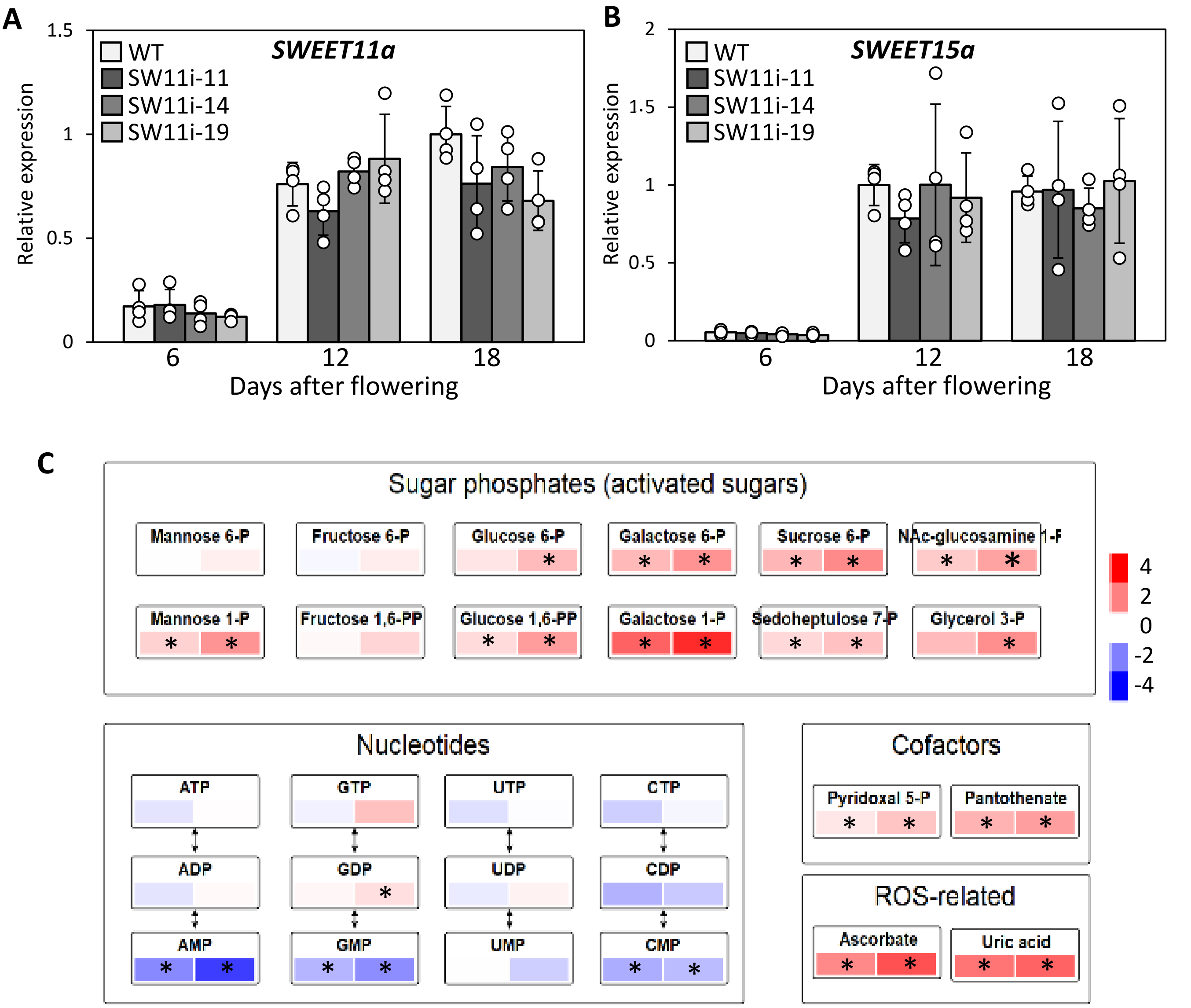

Supplemental Figure S6. Transcript profiles of *HvSWEET11a* and *HvSWEET15a*, and a heatmap showing differences in metabolite abundances in *HvSWEET11b*-repressed grains compared with WT at 12 DAF. (Supports Fig. 3).

(**A**) Expression of *HvSWEET11a* and (**B**) *HvSWEET15a*. Data in (**A**) and (**B**) are shown as the mean; error bars represent standard deviation (n = 4 biological replicates, each consisting of 4-10 caryopses from three spikes). No difference between WT and transgenic lines was found when applying the two-tailed Student's *t*-test. **(C)** Differences in levels of sugar phosphates, nucleotides and cofactors; $\log_2$-fold data are normalized to WT; left and right columns correspond to transgenic lines SW11i-11 and SW11i-19, respectively. *$P < 0.05$ as determined by the Mann–Whitney U-test between WT and the corresponding transgenic line (n = 6 biological replicates, each consisting of 4-10 caryopses from three spikes).

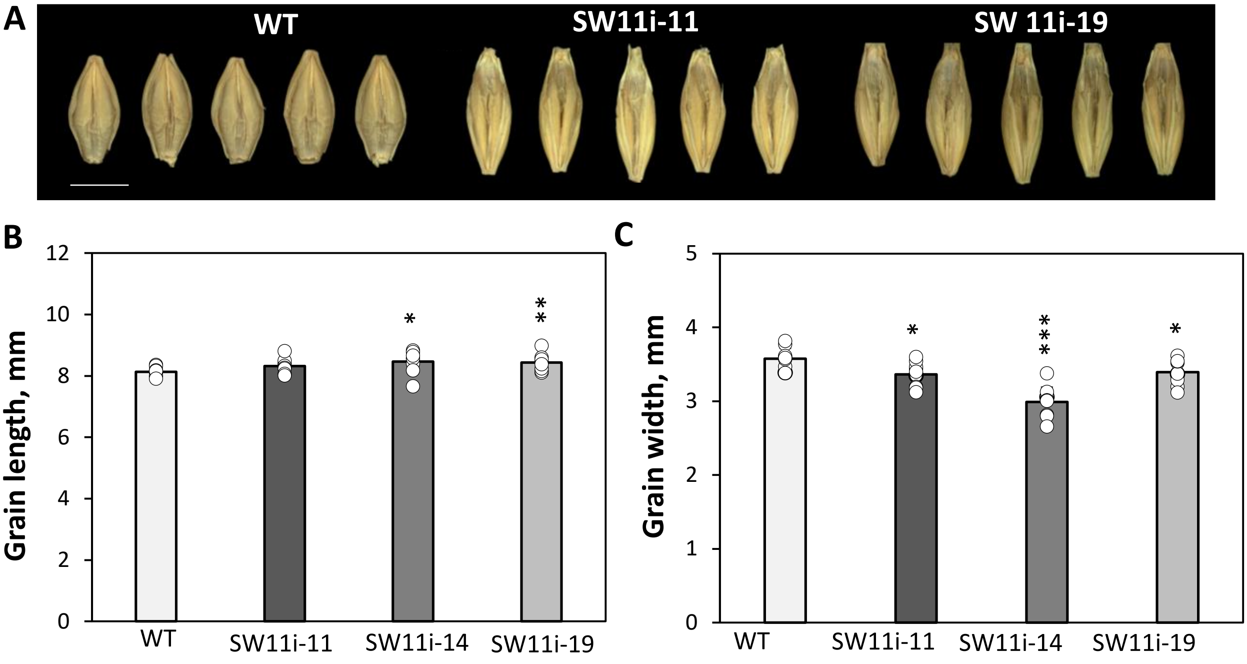

Supplemental Figure S7. **Additional phenotypic characters of *HvSWEET11b*-repressed grains. (Supports Fig. 3 and 5).**

(**A**) A general view of wild-type (WT) and transgenic grains.

(**B**) Grain length.

(**C**) Grain width.

Bars = 5 mm. Values in **B** and **C** are means; error bars represent standard deviation (n = 9 biological replicates, each consisting of ~100 grains). *P < 0.05, **P < 0.01 and ***P < 0.001 as determined by two-tailed Student's t-test between WT and the corresponding transgenic line.

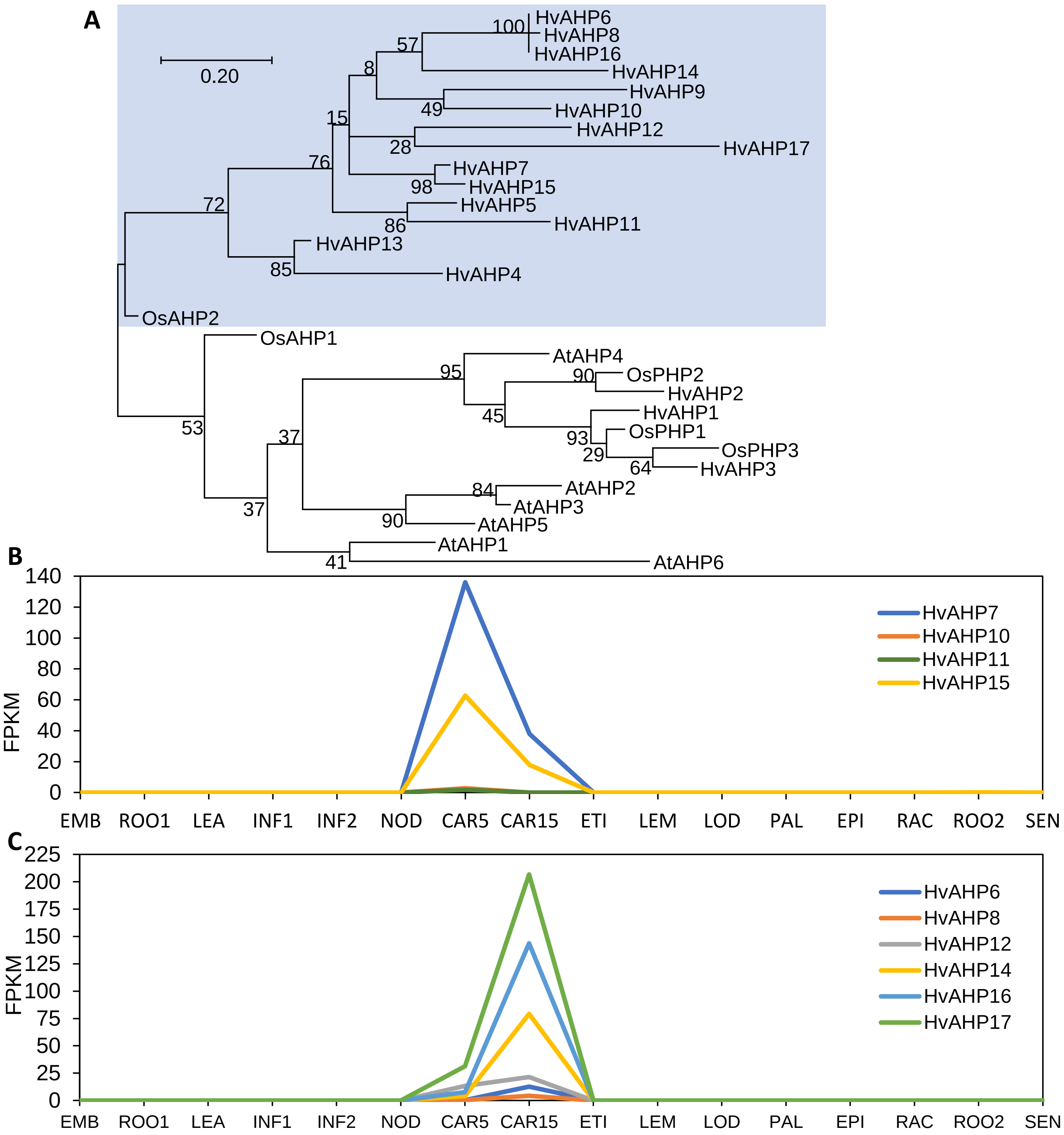

Supplemental Figure S8. The AHP gene family in barley. (Supports Fig. 4).

(**A**) A bootstrap consensus tree of the AHP protein sequences from barley (Hv), rice (Os), and *Arabidopsis thaliana* (At). The maximum likelihood tree was supported by 1000 bootstrap replicates. The scale bar represents evolutionary distances, as quantified by the number of substitutions per amino acid residue. Arabidopsis gene numbers are given by Pareek et al. (2006), and those of rice are from Schaller et al. (2009). Gene IDs for barley and rice are given in Table S1. The blue box highlights the expansion of barley AHP genes at chromosome 4.

(**B, C**) Expression patterns of barley grain-specific AHP genes are predominantly expressed during early development (**B**) and the filling stage (**C**). RNA-seq data were extracted from Monat et al. (2019).

Abbreviations: CAR5, 5-days old developing grain; CAR15, 15-days old developing grain; EMB, 4-days embryo; EPI, epidermal strip; ETI, etiolated seedling; FPKM, fragments per kilobase million; INF1, 5-mm-long developing inflorescences; INF2, 1–1.5 cm developing inflorescence; LEA, seedling shoots; LEM, lemma; LOD, lodicule; NOD, developing tiller; PAL, palea; RAC, rachis; ROO1, seedling root, ROO2, mature root; SEN, senescing leaf.

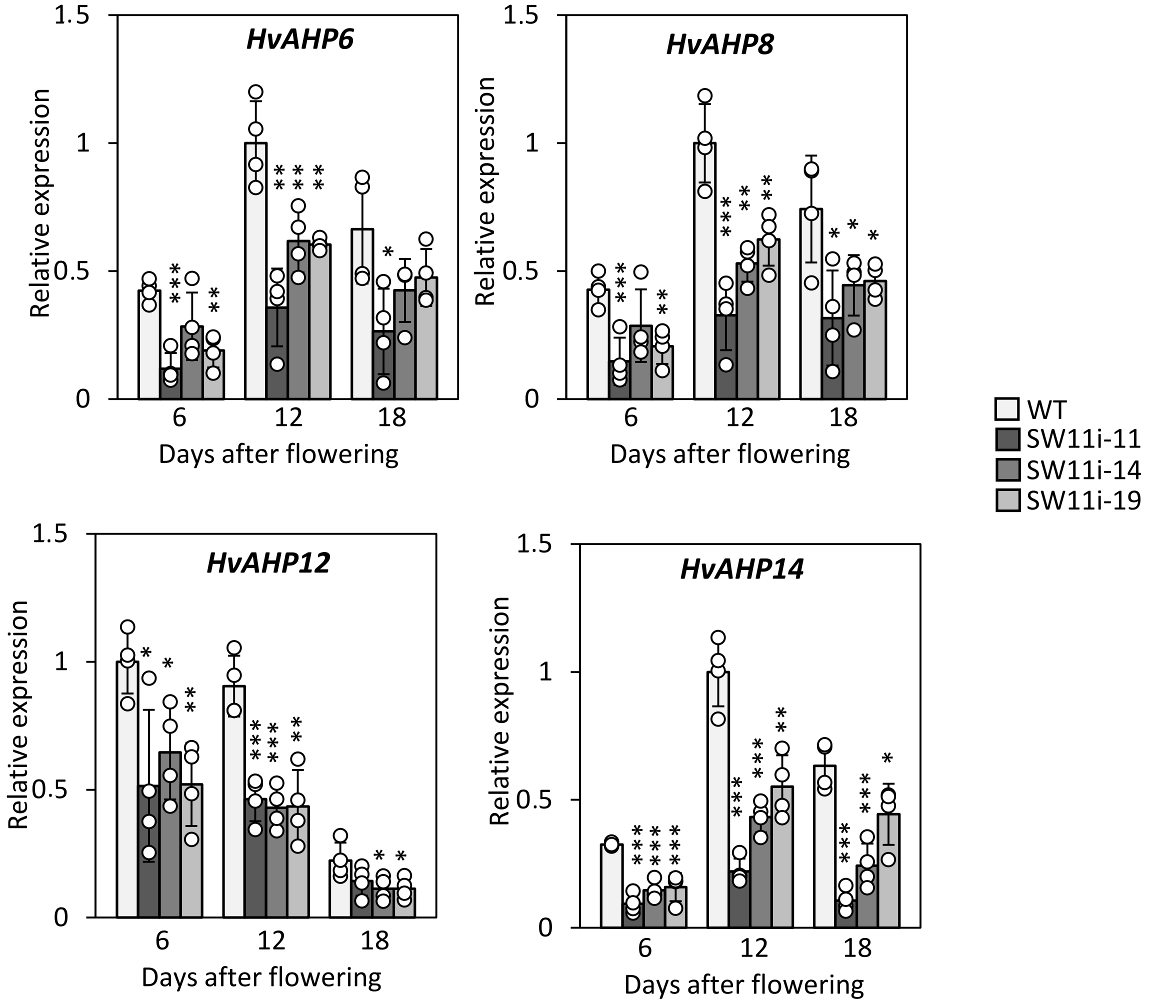

Supplemental Figure S9. Authentic histidine phosphotransferase genes *HvAHP6, HvAHP8, HvAHP12* and *HvAHP14* are downregulated in transgenic *HvSWEET11b*-repressed developing grains compared to the wild-type (WT), as analyzed by qRT-PCR (Supports Fig. 4).
Values are means; error bars represent standard deviation (n = 4 biological replicates, each consisting of 4-10 caryopses from three spikes). *$P < 0.05$, **$P < 0.01$ and ***$P < 0.001$ as determined by two-tailed Student's *t*-test between WT and the corresponding transgenic line.

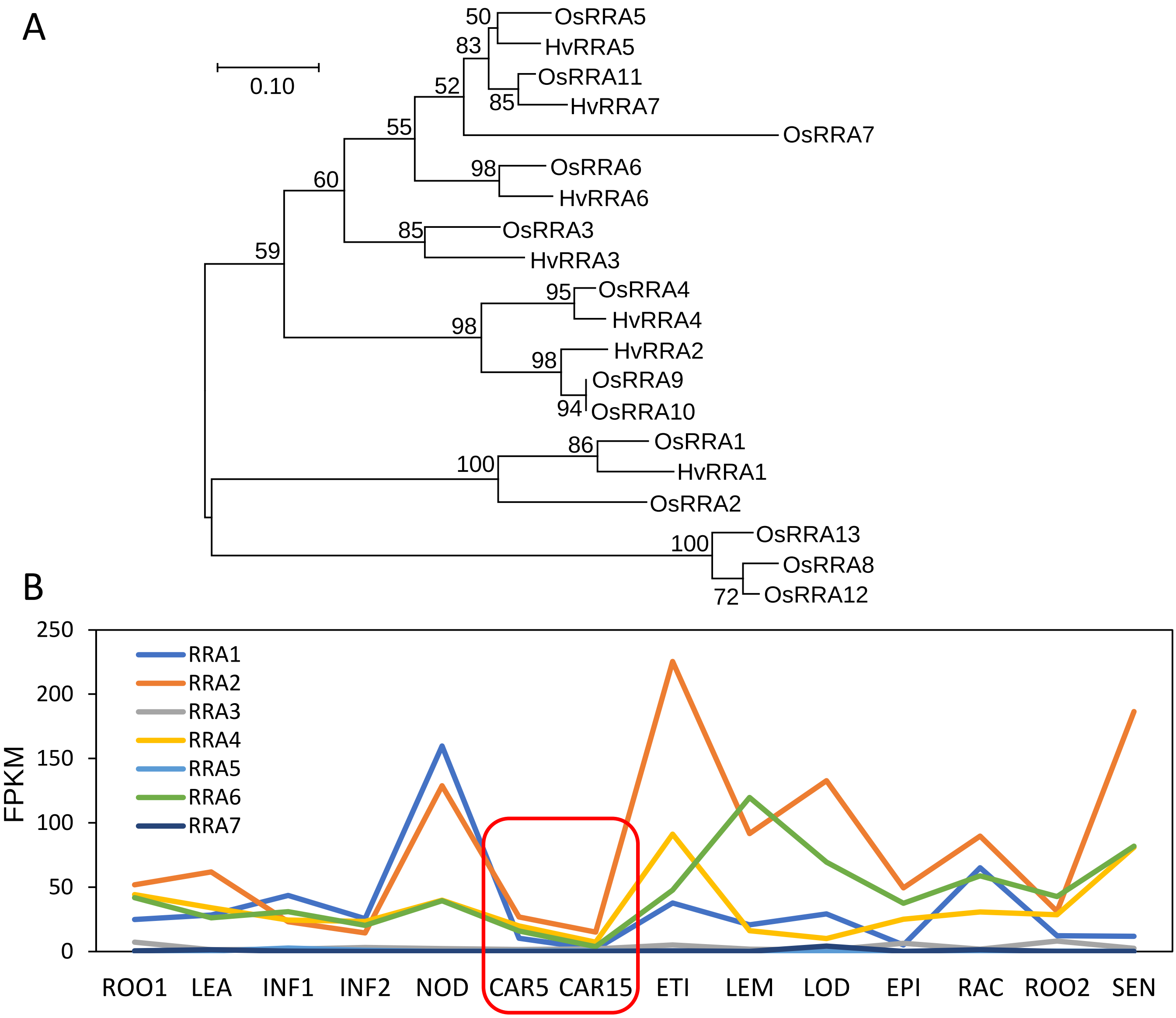

Supplemental Figure S10. The *response regulator A* (*RRA*) gene family in barley. (Supports Fig. 4).

(**A**) A bootstrap consensus tree of the RRA protein sequences from barley (Hv) and rice (Os). The maximum likelihood tree was supported by 1000 bootstrap replicates. The scale bar represents evolutionary distances, as quantified by the number of substitutions per amino acid residue. Rice gene numbers are given by Schaller et al. (2009). Gene IDs for barley and rice are given in Table S1.

(**B**) Expression patterns of barley *RRA* genes extracted from published RNA-seq data (Monat et al., 2019).

Abbreviations: CAR5, 5-days old developing grain; CAR15, 15-day-old developing grain; EPI, epidermal strip; ETI, etiolated seedling; FPKM, fragments per kilobase million; INF1, 5 mm-long developing inflorescences; INF2, 1–1.5 cm developing inflorescence; LEA, seedling shoots; LEM, lemma; LOD, lodicule; NOD, developing tiller; RAC, rachis; ROO1, seedling root, ROO2, mature root; SEN, senescing leaf.

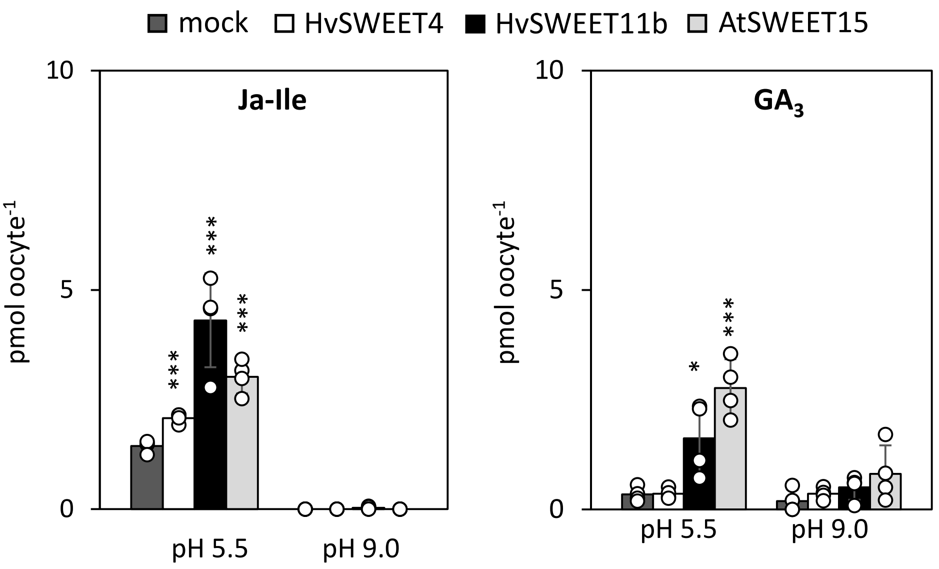

Supplemental Figure S11. Functional analysis of the ability of selected SWEET proteins to transport the mobile forms of jasmonic (Ja-Ile, left) and gibberellic (GA_3, right) acids in *Xenopus* oocytes. (Supports Fig. 4).

The increase in GA_3 and JA-Ile accumulation at pH 5.5 was probably not due to the direct transport activity of these proteins but due to the enhanced diffusion of the compounds at acidic pH in *SWEET*-expressing oocytes compared to mock-treated oocytes. None of the analyzed proteins could transfer the glucosyl ester of abscisic acid (ABA), the transported form of ABA. Values are means; error bars represent standard deviation (n = 4 biological replicates each consisting of individual oocyte). **P* < 0.05, ***P* < 0.01 and ****P* < 0.001 as determined by two-tailed Student's *t*-test between WT and the corresponding transgenic line.

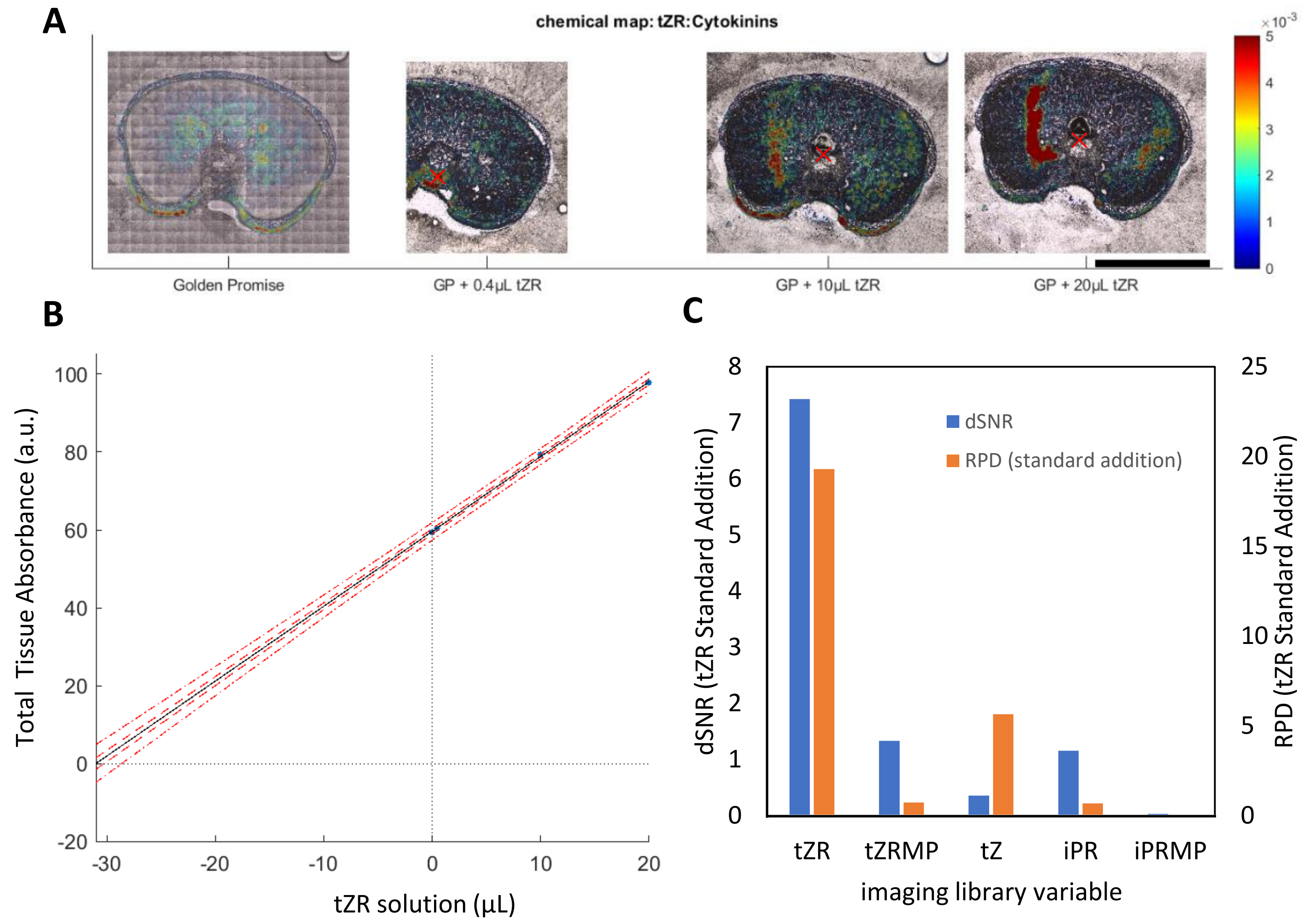

Supplemental Figure S12. **Validation of the *trans*-zeatin riboside measurements by FTIR micro-spectroscopy. (Supports Fig. 4).**

(**A**) Validation of methanolic *t*ZR solution by standard addition of droplets at the marked spot (red X). The tissue acts as a thin layer chromatography solid phase, where methanol spreads in a radial pattern from the application spot (X) outwards through the tissue until evaporation. Methanol soluble compounds, such as *t*ZR, are carried along the methanol front through the tissue, creating a radial front after the application of multiple droplets. (**B**) The sum of the total image absorbance for *t*ZR is plotted against the added *t*ZR standard stock amount to create a standard addition plot for *t*ZR. (**C**) The bar plot shows, in blue, the average signal-to-noise ratio of the additional signal (dSNR) introduced by adding *t*ZR to the tissue. Orange bars show the ratio of performance to deviation (RPD) as a description of the goodness of fit of the standard addition plot. The RPD value represents the ratio of the standard deviation of predicted tissue absorbance against the estimation error of the model. An RPD over 3.5 is generally accepted as quantifiable. Both parameters (dSNR and RPD) show only a high response for *t*ZR images. dSNR values for all other critical cytokinin parameters are below 1.5, representing the detection limit. The RPD values for *t*ZRMP, iPRMP, and iPR are all below a quantifiable margin, indicating that those parameters are unaffected both in the unaltered tissue and when applying additional *t*ZR to the tissue. *t*Z shows a slight bias to *t*ZR (RPD ~5) but is neglectable in its signal intensity since dSNR (< 1.5) of the added signal is below the limit of detection. Bar = 2 mm; color bar represents relative compound tissue absorbance per pixel area.

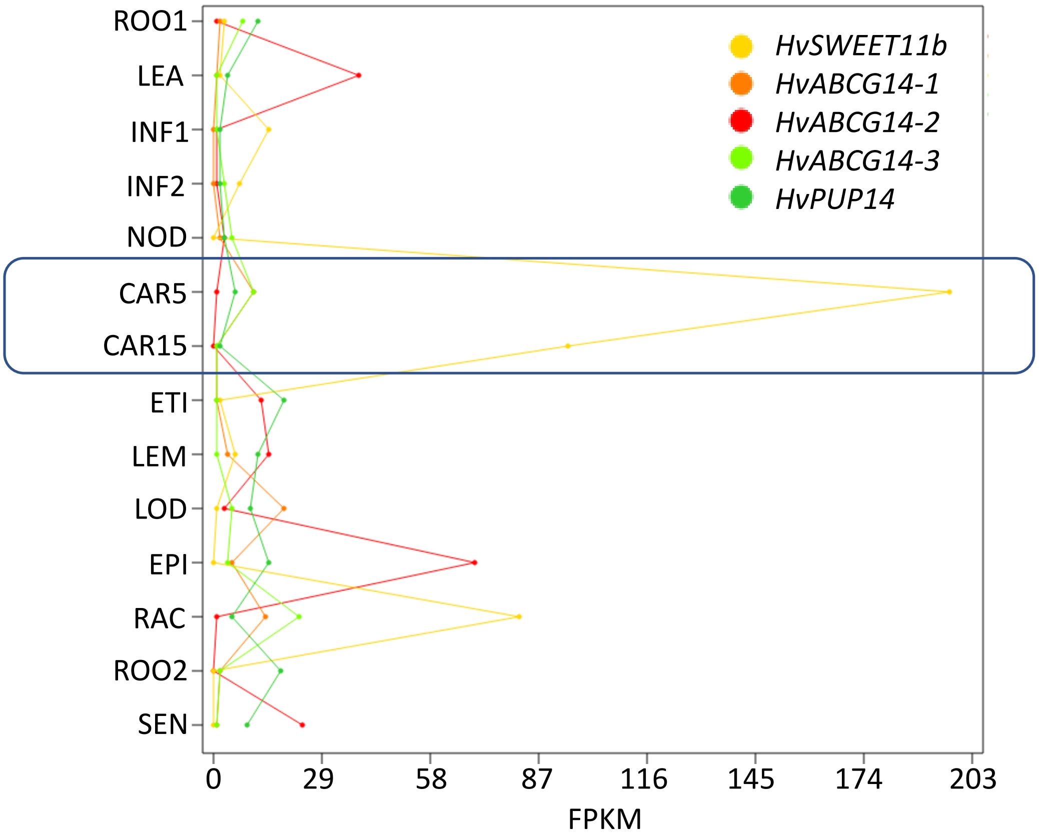

Supplemental Figure S13. The expression of barley orthologs of G-type ABC transporter ABCG14 and purine permease PUP14 compared with SWEET14b in 14 different barley tissues, as revealed by RNA-seq. (Supports Fig. 4).
The data were extracted from Monat et al. (2019). The gene IDs are as follows: *HvSWEET11b* (HORVU.MOREX.r2.7HG0568330.1); *HvABCG14-1* (HORVU.MOREX.r2.4HG0290500.1); *HvABCG14-2* (HORVU.MOREX.r2.4HG0333940.1); *HvABCG14-3* (HORVU.MOREX.r2.7HG0536050.1) and *HvPUP14* (HORVU.MOREX.r2.6HG0501730.1). Abbreviations: ROO1, seedling roots, LEA, seedling shoots; INF1, 5 mm-long developing inflorescences; INF2, 1–1.5 cm developing inflorescences; NOD, developing tillers; CAR5: developing grain at 5 DAF; CAR15: developing grain at 15 DAF; ETI, etiolated seedling; LEM, lemma; LOD, lodicule; EPI, epidermal strips; RAC, rachis; ROO2, mature roots; SEN, senescing leaves.

Supplemental Table S1. MRM transitions for LC-MS/MS analysis.

Analyte	Retention Time [min]	Q1 [*m/z*]	Q3 [*m/z*]	CE [eV]	Reference
tZ $[M+H]^+$	Jan 15	220.0	136.0^{Qt}	15	Ionescu et al., 2017
tZR $[M+H]^+$	Jan 34	352.0	220.0^{Qt}	15	Ionescu et al., 2017
ABA-GE $[M+H]^+$	Jan 60	426.5	274.1^{Qt}	11	This work
		426.5	265.1	4	
iPR $[M+H]^+$	Jan 66	336.1	204.0^{Qt}	15	Ionescu et al., 2017
		336.1	136.0	27	
		336.1	148.0	23	
GA3 $[M-H]^-$	Jan 70	345.2	143.1^{Qt}	25	Tal et al., 2016
		345.2	239.1	12	
		345.2	221.1	21	
JA-Ile $[M-H]^-$	Feb 36	322.0	130.1^{Qt}	17	Ionescu et al., 2017

References

Ionescu IA, López-Ortega G, Burow M, Bayo-Canha A, Junge A, Gericke O, Møller BL, Sánchez-Pérez R (2017) Transcriptome and metabolite changes during hydrogen cyanamide-induced floral bud break in sweet cherry. Front Plant Sci 8: 1233.

Tal I, Zhang Y, Jørgensen ME, Pisanty O, Barbosa IC, Zourelidou M, Regnault T, Crocoll C, Olsen CE, Weinstain R, et al. (2016) The Arabidopsis NPF3 protein is a GA transporter. Nat Commun 7: 11486.

Supplemental Table S2. Chromatographic and mass spectrometry conditions of LC-MS metabolite analyses.

**IC-MS-based untargeted profiling of anionic central metabolites**			**Targeted analysis of cytokinins**		
**Analytical columns:**		**MS-Tune parameters:**	**Analytical columns:**		**MS-Tune parameters:**
Dionex™ IonPac™ AS11-HC-4µm (2x250 & 2x50mm)		Polarity: negative	Column: UPLC CSH C18, 1.7µm (2.1×150 & 2.1x5mm)		Polarity: positive
**Eluents:**		Spray voltage: 3.5 kV	**Eluents:**		Spray voltage: 4 kV
A: H2O		Sheath gas flow: 36	A: H2O + 0.1% (FA)		Sheath gas flow: 30
B: 100mM KOH (generated by Dionex EGC 500 KOH cartridge)		Aux. gas flow: 5	B: ACN + 0.1% FA		Aux. gas flow: 5
**Gradient:**		Capillary temp.: 320 °C	**Gradient:**		Capillary temp.: 320 °C
**Time**	**%B**	Aux. gas temp.: 325 °C	**Time**	**%B**	Aux. gas temp.: 325 °C
0-12	10-25	S-lens: 50	0-5	5	S-lens: 50
12-15	25-100	**Mode: Full MS**	5-15	5-80	**Mode: Targeted-SIM**
15-28	100	Time: 0-30 min	15-15.5	80-100	Time: 0-19 min
28-28.5	100-10	Resolution: 140 K	15.5-17.5	100	Resolution: 70 K
28.5-32	10	m/z Range: 67 - 1000	17.5-18	100-5	Inject time: 200 ms
**Flow parameters:**		Inject time: 200 ms	17.5-20	5	Auto gain control: 5e4
Flow 0.35 mL/min		Auto gain control: 3e6	**Flow parameters:**		Isolation window: 24 m/z
Temp 35 °C		**Mode: Full MS / ddMS2**	Flow 0.5 mL/min		Targeted m/z:
Inj. Vol 10 µL		Time: 0-30 min	Temp 50 °C		21.511.655
		FullMS:	Inj. Vol 2 µL		34.416.409
		Resolution: 70 K			
		m/z Range: 67 - 1000			
		Inject time: 100 ms			
		Auto gain control: 1e6			
		dd-MS2:			
		Resolving power: 17.5 K			
		Inject time: 50 ms			
		Auto gain control: 1e5			
		Loop count: 5			
		Collision energy: 15; 25; 35			
