# Supplementary material for: SWEET11b transports both sugar and cytokinin in developing barley grains
Source: Plant Cell. 2023 Mar 1;35(6):2186–207. doi: 10.1093/plcell/koad055 (PMC10226576; doi:10.1093/plcell/koad055)
Supplement: koad055_Supplementary_Data [file tpc22.00967.PeerReviewReport.pdf › tpc22.00967.PeerReviewReport.pdf]

SWEET11b transports both sugar and cytokinin in developing barley grains

Volodymyr Radchuk, Zeinu M. Belew, Andre Gündel, Simon Mayer, Alexander Hilo, Goetz Hensel, Rajiv Sharma, Kerstin Neumann, Stefan Ortleb, Steffen Wagner, Aleksandra Muszynska, Christoph Crocoll, Deyang Xu, Iris Hoffie, Jochen Kumlehn, Joerg Fuchs, Fritz F. Peleke, Jedrzej J. Szymanski, Hardy Rolletschek, Hussam H. Nour-Eldin, Ljudmilla Borisjuk

Corresponding author: Volodymyr Radchuk, radchukv@ipk-gatersleben.de, and Ljudmilla Borisjuk, borisjuk@ipk-gatersleben.de.

Review timeline:

TPC2022-RA-00392	Submission received:<br>1st Decision:	Aug. 20, 2022<br>Sept. 29, 2022 *manuscript declined*
**TPC2022-RA-00967**	Submission received:<br>1st Decision:	Nov. 29, 2022<br>Dec. 29, 2022 *accept with minor revisions*
**TPC2022-RA-00967R1**	1st Revision received:<br>2nd Decision:<br>Final acceptance:	Jan. 7, 2023<br>Jan. 26, 2023 *acceptance pending, sent to science editor*<br>Feb. 17, 2023

REPORT: (The report shows the major requests for revision and author responses. Minor comments for revision and miscellaneous correspondence are not included. The original format may not be reflected in this compilation, but the reviewer comments and author responses are not edited, except to correct minor typographical or spelling errors that could be a source of ambiguity.)

TPC2022-RA-00392 1st Editorial decision – *declined* **Sept. 29, 2022**

Thank you for choosing to send your manuscript entitled "HvSWEET11b transports both sugar and cytokinin in the developing barley grain" for consideration at The Plant Cell. Your submission has been evaluated by members of the editorial board as well as expert reviewers in your field, and while we are not able to recommend the publication of this manuscript in its current form, we would encourage a resubmission. We have had input from multiple scientists and we have solicited post-review comments.

Reviewer #1 now provides a very elaborate definition of the term biological replicates as it would apply to your oocyte-based transport experiments and we kindly ask you to carefully review this experiment to meet the standards (and definitions) provided there. Reviewer #1 requests that you refrain from considering individual oocytes (from one mother animal) as biological replicates and that you repeat the experiment with at least one additional mother animal to account for the batch-to-batch variation between oocytes originating from different mother animals. Please provide as part of your revised version biological replicates for all experiments in which the sample size is not large enough to have statistical power, as reviewer #1 already outlined and requested in his initial review of the first submission.

Reviewer #2 lists a number of improvements to the manuscript that should substantially improve the paper. Some had already been listed in the review to the first submission and have, unfortunately, not been addressed. We therefore ask you to carefully and thoroughly evaluate the entire manuscript to address all the points raised by reviewer #2.

In addition, reviewer #2 comments that the writing suffers from several problems with grammar and awkward phrasing. We strongly encourage the use of a qualified professional editing service to ensure that the work is presented in the best manner and reaches the broadest possible audience.

-- Reviewer comments:

Reviewer comments on previously declined manuscript and **author responses:**

Reviewer #1:

Radchuk and co-workers present a significantly revised version of their manuscript "HvSWEET11b transports both sugar and cytokinin in the developing barley grain". Generally, the manuscript has benefitted a lot from the review process and as a consequence improved significantly. Most of my issues that primarily addressed the presentation of the data, description of experiments, stats etc. have been addressed nicely.

Thank you very much for reviewing our work, and in particular for your critical comments, which have allowed us to improve some important aspects of the manuscript.

I have one issue left:

The authors argue that oocytes are biological replicates and use the term "biological replicate" now for individual oocytes. This is, in my opinion, wrong and misleading. They argue that "A technical replicate would have been if analyzed the homogenate of the same oocyte several times". This is correct - it would be a technical replicate for the scintillation counting. The individual oocytes in the experiment, as conducted, are technical reps for microinjection and subsequent handling during the efflux experiment. One oocyte is merely one sample in one biological replicate and writing "biological replicate" next to it doesn't make it one. The authors actually acknowledge this since they go on to write: "It is very common that the absolute expression levels vary between batches of oocytes from various frogs, which adds noise to the data analyses. For comparative experiments as the ones presented here it is therefore preferable to conduct the experiment on oocytes from the same frog." This statement is both true and also precisely my concern. This is, in fact, the reason why the vast majority of papers use oocytes from different adult females. Variation in a biological system, such as this, is expected and not a problem. It just means that the number of (true) biological replicates must be increased and the means of these individual experiments on biological replicates should be presented. I realize this is a lot of work but increasing the number of oocytes in one biological rep on the other hand is the definition of p-hacking. The patterns, i.e. the differences between e.g. pH5.5 and pH7.4 for glucose in 2F, will come out identically in another biological rep if the result is robust and the differences are just due to expression level. In a batch of oocytes that expresses worse the difference just come out as 1000 vs 500 instead of 1800 vs 1000 as it is here. If it doesn't that's informative! I realize the paper doesn't hinge on this result but, again from experience, differences like this one may come out differently in another biological rep. This is particularly true in this case because I took the liberty to estimate the statistical power - as I suggested the authors do - and with the effect sizes here n = 6 individual oocytes are the bare minimum to do a meaningful statistic test in the first place- and the numbers here are lower. The other effect sizes are larger so within one biological rep the sample sizes are sufficient within one experiment. Nevertheless, I maintain that repeating experiments with at least one different biological replicate (sic!) is mandatory. In my opinion it's simply good practice and should be standard in a Journal such as Plant Cell to make sure data are repeatable before publication.

We thank the reviewer for sharing his experience with us and for the critical comments we discussed and received additional data (new Fig. S2). We hope that our results are now convincingly presented. In each case, we performed additional (from two to four) repetitions of the experiments with independent batches of oocytes. In particular, ability of HvSWEET11b (and some other barley SWEETs) to transport sugars was proven twice (Fig. 2 and Fig S2), the new described ability of HvSWEET11b to transport cytokinin was checked four times by using different batches of oocytes [Fig. 4D-E (direct assay), 4G-E (competition assay), and Fig S2 (both direct and competition assays)]. To visualize this, we have added the Fig. S2 to the manuscript.

Finally, I agree with the authors that elucidating the transport mechanism is beyond the scope and the effect sizes are so large (actually black and white) in this case that there is no doubt, based on the data presented, about sucrose not inhibiting zeatin uptake and vice versa. I still find this result extremely surprising. Based on the structure and the mechanism we know for this and other facilitators there is no reasonable explanation.

The finding that a SWEET transporter is able to transport cytokinin was surprising to us either. We are intrigued by SWEETs and believe that future work will provide new insights into this topic.

Reviewer #2:

Radchuk et al have greatly improved their manuscript and addressed well the majority of the original reviewer's comments. In particular, I believe that the manuscript benefits from the additional data on the early development of caryopses and pollen viability in the hvsweet11b CRISPR mutant. Altogether, the data supports that the function of the gene is in grain filling and not in gametophyte development or function. It is very important to see that grain development initiates but ultimately fails in the CRISPR mutant. The manuscript also has vast improvements in figure presentation and now it provides the minimum statistical information.

We express our gratitude to the reviewer for the positive feedback on our additional work with the analysis of CRISP mutants and other improvements of the manuscript.

That being said, some easy-to-solve issues remain:

Major

1. I still believe that the authors have not exploited sufficiently their RNAseq data to pull out more cytokinin-regulated genes and support better a reduction of cytokinin signalling in the hvsweet11b RNAi line. Right now, the authors provide only the lower expression of AHPs and a single RR of type A, to support such conclusion. I am aware that the barley genome annotations are quite incomplete but compiling a small list of cytokinin targets based on Arabidopsis or rice literature should not be so hard. Again, this would not require qRT-PCR analysis; the authors should simply look into their own RNAseq data. In principle, other cytokinin-regulated genes should be co-expressed with the AHPs and HvRR2 in that data.

Indeed, barley genome annotation is in its infancy in respect of cytokinin-related genes. Anyway, motivated by the reviewer, we used well described cytokinin-related genes from rice to find barley homologs and track their expression in our RNAseq data (see the table below). As the reviewer suggested, most of detected genes showed tendentially lower expression in *HvSWEET11b*-repressed grains. To re-check these data, we performed qRT-PCRs for several candidates from independent batch of plants and higher number of biological repetitions. We provided such qRT-PCR analyses for several related genes in the main manuscript (Figs 4A, B and S9). Additionally, we selected one gene form the middle of the table (*HvRRB9*) and analysed its expression by qRT-PCR (please see Fig. below). The expression of *HvRRB9* was also decreased in *HvSWEET11b*-repressed grains supporting the RNAseq data. We believe, it is a feasible alternative to confirm the results.

Table. Differences in expression of selected cytokinin-related genes in *HvSWEET11b*-repressed grains when compared to wild type as defined by RNAseq analysis ("-" means repression).

				6 DAF			12 DAF		
rice gene ID	rice gene annotation	barley gene ID	barley gene annotation	logFC	P.Value	Padj	logFC	P.Value	Padj
Os03g49500	OsERS1	HORVU4Hr1G006260	Ethylene receptor, ERC1b	**-0,35**	0,052	0,496	**-0,06**	0,718	0,999
Os03g49500	OsERS1	HORVU5Hr1G119650	Ethylene receptor, ERC1c	**-1,08**	4E-04	0,052	**-0,35**	0,178	0,999
Os04g08740	OsETR2	HORVU2Hr1G000030	Ethylene receptor 2a, ETR2a	**-2,47**	0,194	0,731	**-0,54**	0,753	0,999
Os04g08740	OsETR2	HORVU0Hr1G001750	Ethylene receptor 2b, ETR2b	**-0,51**	0,09	0,585	**-0,55**	0,079	0,999
Os06g44410	OsHK1	HORVU7Hr1G111360	histidine kinase 1a, HK1a	0,457	0,333	0,819	**-1,35**	0,122	0,999
Os02g50480	OsCHK2	HORVU6Hr1G077070	histidine kinase 3	**-0,27**	0,653	0,941	**-0,21**	0,769	0,999
Os04g36070	OsRRA1	HORVU2Hr1G077000	Two-component response regulator RRA1	0,449	0,151	0,681	**-0,51**	0,26	0,999
Os02g55320	OsRRB3	HORVU6Hr1G085560	Two-component response regulator RRB3	**-0,79**	0,029	0,41	**-0,33**	0,365	0,999
Os02g08500	OsRRB4	HORVU6Hr1G028680	Two-component response regulatorRRB4	**-0,31**	0,35	0,826	**-0,56**	0,136	0,999

Os02g08500	OsRRB4	HORVU6Hr1G087520	Two-component response regulator-like	**-0,47**	0,199	0,735	**-1,4**	0,059	0,999
Os04g28130	OsRRB9	HORVU6Hr1G087540	Two-component response regulator RRB9	**-0,67**	0,091	0,589	**-0,6**	0,399	0,999
Os08g35670	OsRRB13	HORVU7Hr1G118290	Two-component response regulator RRB13	**-0,31**	0,315	0,81	**-0,47**	0,539	0,999
Os08g35670	OsRRB13	HORVU7Hr1G114130	Two-component response regulator RRB16	**-0,41**	0,486	0,883	0,587	0,522	0,999
Os03g53100	OsRRC1	HORVU5Hr1G109070	Two-component response regulator RRC6	**-0,18**	0,384	0,836	**-0,52**	0,526	0,999
Os04g13480	OsRRC2	HORVU3Hr1G034530	Two-component response regulator RRC7	**-0,46**	0,272	0,789	**-0,8**	0,453	0,999
Os04g13480	OsRRC2	HORVU3Hr1G034520	Two-component response regulator RRC8	**-0,8**	0,017	0,34	**-0,59**	0,425	0,999
Os04g13480	OsRRC2	HORVU3Hr1G034510	Two-component response regulator RRC9	**-0,64**	0,043	0,471	**-0,54**	0,323	0,999
Os11g05930	OsRRR4	HORVU4Hr1G021010	Two-component response regulator RRR4	0,177	0,703	0,954	**-0,41**	0,409	0,999

As mentioned above, we are performing new experiments with the aim to elucidate the cytokinin-transporting role of HvSWEET11b in more details, which however will be part of future publication.

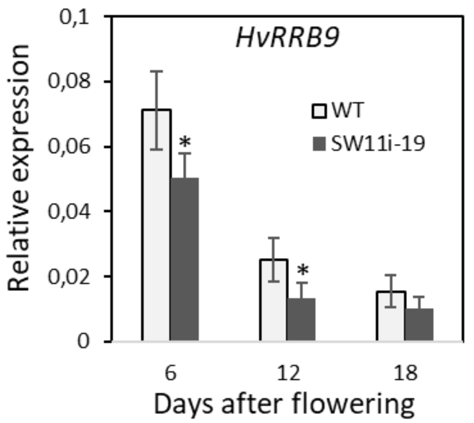

Figure. Expression of *HvRRB9* in wild type and *HvSWEET11b*-repressed line SW11i-19 (used in RNAseq) during grain development. Data are shown as the mean; error bars represent standard deviation (n = 4 biological replicates, each consisting of 4-10 caryopses from three spikes). *P < 0.05 as determined by two-tailed Student's *t*-test between wild type and the transgene line.

2. Line 200. "HvSWEET11b was weakly expressed in the anther". Please remove the adjective 'weakly'; the expression of the gene in anthers seems in the same order of magnitude as in grains (Fig. S1A) but in the grains they call this 'highly abundant' (line 136). Use the same criteria for both data! I also see that in their response to my comments, the authors consider the expression in anthers 'negligible' because they compare it to the microdissected tissues analyzed in Fig. S1B. That is not a fair comparison because expression is obviously enriched in the microdissected tissue.

We agree with proposed wording and changed these positions in the text.

3. Lines 202-3. "... mutation in HvSWEET11b probably did not have a strong effect on pollen development". Remove the adjective 'strong'. The evidence simply supports that the mutation has no effect in pollen development. No data supports a 'quantitative' phenotype.

Corrected.

4. Fig. S3D. I cannot find anywhere in the text what kind of 'vitality' stain the authors used (was it Alexander's?). They need to state in the legend the actual stain or dye used and the protocol should be described or cited in the Materials and methods.

We used staining protocol after Peterson et al. (2010). We added this information to the Material and Method section, into References and mentioned in the legend to renumbered Fig. S4D.

5. Line 242, figure 3E. Remove UDP-glucose from the sentence. The data does not show significant difference between WT and the RNAi lines for that metabolite. It is not possible to conclude that it is elevated in the RNAi lines.

Done.

6. Line 243. The 6-fold increase in trehalose 6-phosphate is seen on average in only one of the two RNAi lines. I suggest to remove "6-fold" and simply state that the levels were higher than in the wild-type in the two lines.

Done.

7. Fig. S10. Authors must adapt the y-axis scale to the maximum level observed in the data, just like they do in Fig. 4E. Moreover, please refrain from calling the transport activity observed at pH 5.5 "very low". Suppl. Dataset 1 shows that the average transport of JA-Ile at pH 5.5 is in the same order of magnitude as the transport of some cytokinins presented in fig. 4D-F. However, in that case the authors do claim that there is transport of those cytokinins while for JA-Ile they engage into an obscure explanation of 'simple diffusion'. I do not believe that this is appropriate: First, the authors should use the same measure for all their data; and second, they should refrain from any speculative explanation if not based on literature or common physical chemistry knowledge.

We thank Reviewer for advice. According to Reviewer recommendation: (i) we adapted the y-axis in the new Fig. S11; (ii) the words "very low" are omitted in the main text. In current version the Fig.S10 is Fig.S11.

8. Lines 350-351. "tZR was (...) decreased in the endosperm of transgenic grains compared with wild-type". Authors do not provide quantification to support this statement and I do not think that the panel in fig. 1K is sufficient. Thus, please remove any statement about lower tZR levels in the endosperm. Also, a minor point: authors should cite fig. 1K in line 351.

We agreed that we did not quantified the tZR in the endosperm and therefore took away this statement. The Fig. 1k does not exist in the manuscript. If the reviewer meant Fig. 4K, we placed its citation to the correct position in the text.

9. The authors claim that professional English editors have checked the current version of the manuscript. However, many obvious (and easy-to-spot) problems persist. Some wrong sentences read exactly as in the first version and others that were fine, are now wrong! An example, line 420: "Five of the ten barley SWEET genes are likely to encode sucrose transporters (HvSWEET11a, HvSWEET11b, HvSWEET14a, HvSWEET14b, and HvSWEET15a) are transcribed in the developing grain".

We restored the first version of the sentence.

10. I believe reviewer 1 asked which time points the authors used to collect the transport competition data reported in Fig. 4G-H. However, the authors have failed to provide this information (they mistakenly thought that the reviewer was referring to fig. 2 only).

We apologize for any misunderstandings. We have performed additional work and include the data in Fig. S2, which now support Fig. 4.

11. In my comments to the first submission, I stated a mistake in the legend to panel 5F, which is still there. I guess I have to spell it letter by letter so that the authors understand. They write: "The red lines indicate the greater length of the WT grains, resulting from their formation of a longer husk.". This is incorrect, the red lines refer to the grains of the RNAi lines, not the WT grains. And on a side note, "silenced" is a valid synonym for the reduction in gene expression caused by RNAi. So, sorry, but yes, the authors did use silenced lines in the entire work.

Many thanks for clarifying our mistake. Clear, transgenic grains were longer. We corrected this sentence in the legend to Fig. 5F.

Minor issues

12. It is great that the authors added new text to the abstract to make it more informative with the grain phenotypes of the RNAi lines. However, the new text is a bit obscure: "The transgenic effect unfolded during the main seed filling phase; the transgenic grain became smaller but almost reassembled with the wild-type morphological integrity and composition (phenotype)". I do not even understand what they mean by 'reassembled'. I suggested to simply use my summary sentence of their work: "Lower expression of HvSWEET11b in developing grains reduced overall size, sink strength, the number of endopolyploid endosperm cells and the content of starch and protein". Of course, the authors are free to decide what they think is best.

We agree that the wording proposed by the reviewer is clearer and used this in the abstract.

13. Figure 1S. "etc" not defined in legend.

Done.

14. Figure 2E. Maybe it is just me but the use of color and shade to distinguish the four experimental set ups was quite confusing! Why not use the same color for the 'treatment' (i.e. mock vs. HvSWEET11b) and the shade difference for 'external' vs. 'internal'? Or perhaps use different glyphs (circles vs. triangles) to distinguish the 'treatments'.

We agree that using different line colours (black/grey) and different glyphs (circle/diamond) increased readability of the Fig. 2E. We made corresponding corrections.

15. Line 259. Sentence is incorrect. It was fine in the previous version.

We did not find any changes in earlier and later version at this position.

16. Suppl. Dataset 1 for Fig. 4D-F and S10: In column D, the name of the transporters at pH9 is wrong; authors used the name of the RNAi lines.

Thank you for noticing this error, we are correcting it gratefully.

TPC2022-RA-00967 1st Editorial decision – *accept with minor revisions* Dec. 29, 2022

We have evaluated your revised manuscript entitled "HvSWEET11b transports both sugar and cytokinin in the developing barley grain." Note that at this stage, we felt that editorial evaluation without input from external reviewers would be sufficient. On the basis of our evaluation, the board of reviewing editors would like to accept your manuscript for publication in The Plant Cell. This acceptance is contingent on revision based on the comments of our editors. In particular, please consider the following:

1) l. 125 - 130 - Please provide a reference for the individual statements (Figure, publication)

2) All graphs shown in the paper should be harmonized and many can be graphically improved, individually, and in their arrangement with the other panels (e.g. in Figure 2F, glucose is fused with the lines framing the graph etc.). Please see the instructions at the Plant Cell web page if you are insecure about how to proceed.

3) Figure 1B and C - E; in C - E, most likely the colour code of the bars and the sample ID corresponds to the colour code introduced in B. Otherwise this cannot be explained. There appear, however, some inconsistencies (blue appears twice in E at 14 days; the order of colours differs between B and C - E etc.). This figure should be checked carefully and an explanation of the colour code should be provided in the legend. As mentioned above, this should also taken as an opportunity to graphically improve this figure and the graphs.

4) l. 163 - "a weak ability (to transport sucrose)" needs to be precised better

5) Please also consider providing a graphically more appealing Figure 8.

-- Reviewer comments:

TPC2022-RA-00967R1 1st Revision received Jan. 7, 2023

Reviewer comments on previous submission and **author responses**:

Editor comments:

1) l. 125 - 130 - Please provide a reference for the individual statements (Figure, publication)

We added two references to the text (line numbers 125 – 130): Unlike their orthologs in rice (Yuan et al., 2014) and A. *thaliana* (Chen et al., 2012), SWEET11, SWEET13, SWEET14, and SWEET15 are each represented in barley by multiple (two or three) genes, rather than by a single copy.

2) All graphs shown in the paper should be harmonized and many can be graphically improved, individually, and in their arrangement with the other panels (e.g. in Figure 2F, glucose is fused with the lines framing the graph etc.). Please see the instructions at the Plant Cell web page if you are insecure about how to proceed.

All figures have been checked and many details have been improved. The modifications include clarification of colour coding (Figures 1B and C - E), the elimination of word fusion with lines and frames in graphics (Figure 2 and other figures), graphical improvement (Figure 1B,C,E) and an improvement in visual appeal (Figure 8). All comments have been considered, and figures have been harmonised.

3) Figure 1B and C - E; in C - E, most likely the colour code of the bars and the sample ID corresponds to the colour code introduced in B. Otherwise this cannot be explained. There appear, however, some inconsistencies (blue appears twice in E at 14 days; the order of colours differs between B and C - E etc.). This figure should be checked carefully and an explanation of the colour code should be provided in the legend. As mentioned above, this should also taken as an opportunity to graphically improve this figure and the graphs.

Reviewers are absolutely right, "…*the colour code of the bars and the sample ID corresponds to the colour code introduced in B*". Unfortunately, blue (shading) was not the optimal for color-coding. To answer the reviewers' criticism "…*the order of colours differs between B and C* " we replace this one by other (contrasting) colour and use it accordingly in C- E. Therewith misleading labelling was eliminated. In addition, we have introduced one short sentence to the figure legend, please see Figure 1B: The tissue color code corresponds to the bars in C-E.

4) l. 163 - "a weak ability (to transport sucrose)" needs to be precised better.

We processed the statement by adding following words (to transport sucrose) in the sentence (line 161-163).

5) Please also consider providing a graphically more appealing Figure 8.

We have improved the figure and hope that this is more appealing.

Please highlight all changes and include a detailed annotation of changes of the text, with line numbers, and noting your responses to the comments.

There are two changes in the main text (line 125-130) and (161-163). There is one change in legend to Figure (1B), where short sentence is added: The tissue color code corresponds to the bars in C-E. All changes (text and legend) are highlighted in red.

TPC2022-RA-00967R1 2nd Editorial decision – *acceptance pending* Jan. 26, 2023

We are pleased to inform you that your paper entitled "HvSWEET11b transports both sugar and cytokinin in the developing barley grain" has been accepted for publication in The Plant Cell, pending a final editorial review by a science editor.

Final acceptance from Science Editor Feb. 17, 2023
